# Evaluating the Impact of Physical Activity Apps and Wearables: Interdisciplinary Review

**DOI:** 10.2196/mhealth.9054

**Published:** 2018-03-23

**Authors:** Claire McCallum, John Rooksby, Cindy M Gray

**Affiliations:** ^1^ Institute of Health and Wellbeing University of Glasgow Glasgow United Kingdom; ^2^ School of Computing Science University of Glasgow Glasgow United Kingdom

**Keywords:** mobile health, physical activity, smartphone, fitness trackers, wearable electronic devices, research design, evaluation studies as topic, efficiency

## Abstract

**Background:**

Although many smartphone apps and wearables have been designed to improve physical activity, their rapidly evolving nature and complexity present challenges for evaluating their impact. Traditional methodologies, such as randomized controlled trials (RCTs), can be slow. To keep pace with rapid technological development, evaluations of mobile health technologies must be efficient. Rapid alternative research designs have been proposed, and efficient in-app data collection methods, including in-device sensors and device-generated logs, are available. Along with effectiveness, it is important to measure engagement (ie, users’ interaction and usage behavior) and acceptability (ie, users’ subjective perceptions and experiences) to help explain how and why apps and wearables work.

**Objectives:**

This study aimed to (1) explore the extent to which evaluations of physical activity apps and wearables: employ rapid research designs; assess engagement, acceptability, as well as effectiveness; use efficient data collection methods; and (2) describe which dimensions of engagement and acceptability are assessed.

**Method:**

An interdisciplinary scoping review using 8 databases from health and computing sciences. Included studies measured physical activity, and evaluated physical activity apps or wearables that provided sensor-based feedback. Results were analyzed using descriptive numerical summaries, chi-square testing, and qualitative thematic analysis.

**Results:**

A total of 1829 abstracts were screened, and 858 articles read in full. Of 111 included studies, 61 (55.0%) were published between 2015 and 2017. Most (55.0%, 61/111) were RCTs, and only 2 studies (1.8%) used rapid research designs: 1 single-case design and 1 multiphase optimization strategy. Other research designs included 23 (22.5%) repeated measures designs, 11 (9.9%) nonrandomized group designs, 10 (9.0%) case studies, and 4 (3.6%) observational studies. Less than one-third of the studies (32.0%, 35/111) investigated effectiveness, engagement, and acceptability together. To measure physical activity, most studies (90.1%, 101/111) employed sensors (either in-device [67.6%, 75/111] or external [23.4%, 26/111]). RCTs were more likely to employ external sensors (accelerometers: *P*=.005). Studies that assessed engagement (52.3%, 58/111) mostly used device-generated logs (91%, 53/58) to measure the frequency, depth, and length of engagement. Studies that assessed acceptability (57.7%, 64/111) most often used questionnaires (64%, 42/64) and/or qualitative methods (53%, 34/64) to explore appreciation, perceived effectiveness and usefulness, satisfaction, intention to continue use, and social acceptability. Some studies (14.4%, 16/111) assessed dimensions more closely related to usability (ie, burden of sensor wear and use, interface complexity, and perceived technical performance).

**Conclusions:**

The rapid increase of research into the impact of physical activity apps and wearables means that evaluation guidelines are urgently needed to promote efficiency through the use of rapid research designs, in-device sensors and user-logs to assess effectiveness, engagement, and acceptability. Screening articles was time-consuming because reporting across health and computing sciences lacked standardization. Reporting guidelines are therefore needed to facilitate the synthesis of evidence across disciplines.

## Introduction

Physical inactivity is a major public health problem [1], with 23% of adults worldwide not meeting recommended levels of physical activity (only 35% and 40% in the United States and the United Kingdom, respectively [2]). Many smartphone apps and wearables designed to improve physical activity are available. They often use data from in-device sensors to provide self-monitoring and feedback [3]. The potential of apps and wearables to increase physical activity and ultimately improve health outcomes, such as management of cardiovascular disease, obesity, and type 2 diabetes, has been widely recognized [4-9]. However, evaluating the impact of physical activity technologies can be challenging, because of the rapid rate at which they evolve [10-12]. Randomized controlled trials (RCTs), the “gold standard” of effectiveness evaluations, can take several years to conduct [11] and require interventions to be stable and unchanged throughout this period [12]. Consequently, researchers have emphasized the need for greater “efficiency” (ie, rapid, responsive, and relevant [11], or agile [13] research) when evaluating mobile health (mHealth) technologies.

Evaluating the effectiveness of mHealth technologies can be particularly challenging because of their “complexity” [14]. Physical activity apps and wearables often contain multiple components, which can interact with context and produce different outcomes for different people in different settings [15,16]. To understand overall effectiveness, studies should evaluate real-world engagement with, and response to, an intervention [17]. Measuring these factors alongside effectiveness can help interpret and explain variation in effectiveness outcomes, (ie, *why* the intervention worked or did not work [16-19]). Accordingly, mHealth researchers have been encouraged to assess “engagement” and “acceptability” [14,20]. However, how to define and distinguish these constructs is still a subject of debate; for example, some digital health researchers have conceptualized engagement as a behavioral construct [21,22], whereas others propose that it is composed of both behavioral and subjective components [20,23]. The latter view produces overlaps between engagement and acceptability, and therefore for clarity during this review, we define “engagement” as users’ interaction and usage behavior (ie, a purely behavioral construct), and “acceptability” as users’ subjective perceptions and experiences.

To increase the efficiency of mHealth evaluations, particular research designs and data collection methods have been recommended [11,14,24,25]. Single-case designs or “n-of-1” studies, in which participants serve as their own control, may be conducted relatively quickly and easily using mHealth technology [13,26]. To evaluate overall effectiveness, the Continuous Evaluation of Evolving Behavioral Intervention Technologies was developed to test multiple versions of an app simultaneously [27]. To test the impact of individual components, quick factorial approaches have been developed, including the multiphase optimization strategy (MOST), which rapidly tests many experimental conditions [28,29], and Sequential Multiple Assignment Randomized Trials [30] and micro-randomized trials [31], which both evaluate components that adapt across time.

To improve the efficiency of data collection, researchers can capitalize on the technological capabilities of consumer devices. In-device sensors (ie, accelerometers, gyroscopes, and other sensors embedded in smartphones and wearables) can be used to measure outcomes objectively [24,26]. Their internet connectivity and ability to collect continuous, high-density data remotely can improve efficiency over other “intermittent and limited” methods [24], such as questionnaires and traditional pedometers. Smartphones and wearables can also automatically record user interactions and app use [20]. Human computer interaction (HCI) researchers have used such device-generated logs to measure engagement objectively and remotely [32,33]. Log data has also been used for exploring acceptability, when used alongside qualitative methods [33].

Recommended evaluation designs and methods, as well as multidisciplinary approaches, may advance mHealth research [10,25]. Yet, a recent review of registered clinical trials found that evaluations of mHealth apps targeting a range of clinical conditions did not use either rapid research designs or innovative data collection methods [34]. The authors recommended that future reviews should incorporate a broader set of studies beyond those on ClinicalTrials.gov to identify rapid research designs.

The study team aimed to investigate, across health and HCI disciplines, the extent to which evaluations of physical activity apps and wearables (1) use recommended rapid research designs; (2) assess engagement and acceptability as well as effectiveness; and (3) employ efficient data collection methods (ie, in-device sensors and device-generated logs). The team also aimed to explore those dimensions of engagement and acceptability that are assessed.

## Methods

### Study Design

The study team conducted an interdisciplinary scoping review of the research designs, objectives, and data collection methods used in evaluations of physical activity apps and wearables. Scoping reviews are used to rigorously and comprehensively map the range of research activities undertaken in an emerging field [35]. In accordance with scoping review methodology [36], the team did not assess quality or reject studies on the basis of research design, as this would have excluded many HCI studies. The team adapted the framework suggested by Arksey & O’Malley [35] and Levac et al [37], to include 4 steps (1) identification of relevant articles; (2) study selection; (3) charting and extraction of the data; and (4) collation, summarization, and reporting of results.

### Identification of Relevant Articles

An initial literature search of 8 databases was conducted between August to September 2015 and updated in March 2017. These included 3 health and clinical databases (PubMed, PsycINFO, and Web of Science), 4 computing science databases (Association for Computing Machinery Digital Library (ACM), Institute of Electrical and Electronics Engineers (IEEE), Springer and Science Direct) and 1 interdisciplinary database (mHealth Evidence). The search terms used for different database are presented in Textbox 1. Articles were restricted to English language. No time limit was specified. Protocols, conference proceedings, and extended abstracts were all eligible. The reference lists of systematic reviews were hand-searched for further relevant articles.

### Study Selection

Studies were included if they evaluated mobile technologies that provided sensor-based feedback on physical activity. To describe the full range of data collection methods used to measure physical activity, studies using objective and self-report measures were both included. Exclusion criteria were (1) no empirical data was collected (ie, systematic or methodological reviews, position papers and articles that only described technologies); (2) physical activity was not measured (ie, studies measured only sedentary time, activity skills, and gait); (3) the study only evaluated sensor or algorithmic performance (ie, accuracy in recognizing or classifying physical activity); (4) the sensor was not mobile; (5) the only mobile technology used was a pedometer without the capacity to connect to another device or the internet (this exclusion criterion was included to focus the review on wearable devices with more advanced feedback capabilities than standard pedometers).

All abstracts and full-text articles were reviewed by CM, and 5% of abstracts were independently reviewed by CG or JR. Discrepancies were discussed by the 3 authors, and all were resolved. Any articles representing the same study were merged.

### Data Extraction

A data extraction form was developed to include (1) study characteristics (ie, publication year, country of study, number of participants, age of participants, study duration, whether a protocol or full trial); (2) research design details (ie, experimental or nonexperimental design, number of groups, experimental or control group details, randomization), and intervention characteristics (ie, technologies or devices used to deliver intervention, key intervention features); (3) research objectives and outcomes measured; (4) analyses undertaken (descriptive, inferential, thematic); and (5) data collection methods used (eg, in-device or external sensors, user-logs, questionnaires, interviews, focus groups). All reviewers independently extracted 5 papers (5%) to ensure consistency and reliability of data extraction.

### Collation, Summarization, and Reporting of Results

The study team adopted a mixed-methods descriptive approach to analyze the extracted data [35]. The team first calculated frequencies in relation to study characteristics and each research design identified and mapped intervention characteristics (ie, the components or app features that studies evaluated). Next, the research objectives and outcomes that studies measured, as reported by authors, were used to categorize studies according to whether they investigated effectiveness (ie, changes in physical activity). Categorizing studies according to whether they investigated engagement and acceptability required a more iterative approach, as definitions of these constructs are less widely agreed. Working definitions of engagement (ie, user interaction with the device or usage behavior) and acceptability (ie, users’ subjective perceptions and experiences) were applied to extracted research objectives, outcome measures, and data collection methods to develop a series of broad codes in relation to engagement (ie, engagement, usage, use, adherence, compliance) and acceptability (ie, acceptability, satisfaction, user experience, usability). These codes were applied to all studies to allow them to be categorized according to whether they investigated engagement and/or acceptability. Frequencies are reported for the number of studies in each category.

**Search terms used in the scoping review.**

**Health and Clinical Databases: PubMed, Web of Science, PsycINFO**

Exercise/physical activity/physical activities

AND mobile/mobile phone/smartphone/sensor/smart watch/ wearable/wearable device

AND intervention/program/app/application

AND evaluate/evaluation/ assessment/measure/trial/test MeSH terms (PubMed only): “motor activity”, “exercise”, “cellular phones” and “studies with evaluation as topic”

**Computing Science Databases: ACM, IEEE, Springer, Science Direct**

Physical exercise/physical activity/physical activities

AND mobile/“mobile phone”/smartphone/sensor/smartwatch/wearable/wearable device/ubiquitous computing

AND intervention/program/app/application/activity tracking/personal informatics

AND evaluate/evaluation/assessment/measure/trial/test

**Interdisciplinary Database: mHealth Evidence**

Physical activity/physical exercise

In relation to effectiveness, the team calculated the proportion of studies that used only descriptive statistics (as opposed to inferential statistical analysis) and grouped studies that used sensors to collect physical activity data according to whether they used in-device sensors or external sensors (ie, additional, validated devices). The team then calculated frequencies for the data collection methods used in each group, and a chi-square test of independence was conducted to examine whether the type of sensor used was related to the type of research design using R statistical software (RStudio, version 1.0.136).

In relation to engagement and acceptability, the data collection methods extracts were first used to calculate frequencies in relation to the data collection methods studies employed (eg, user-logs, questionnaires, focus groups, interviews). Each extract was then read carefully to identify detailed subcodes that described the different elements assessed for each construct (ie, any specific behaviors logged, questionnaire items used, or interview or focus group topics described), and the One Sheet of Paper method [38] was used to generate broad dimensions of engagement and acceptability by grouping these subcodes according to their similarity.

A random sample of all studies (20.7%, 23/111) was independently coded (by CG) to improve rigor in categorizing studies and generating the dimensions in relation to engagement and acceptability; discrepancies were discussed and consensus was reached on the final dimensions. Discussions suggested that some of the dimensions initially associated with acceptability were specifically related to the properties of the app or device and therefore did not relate to acceptability per se. These dimensions were retained and categorized as “usability.”

## Results

### Summary of Search Results

**Figure 1:**
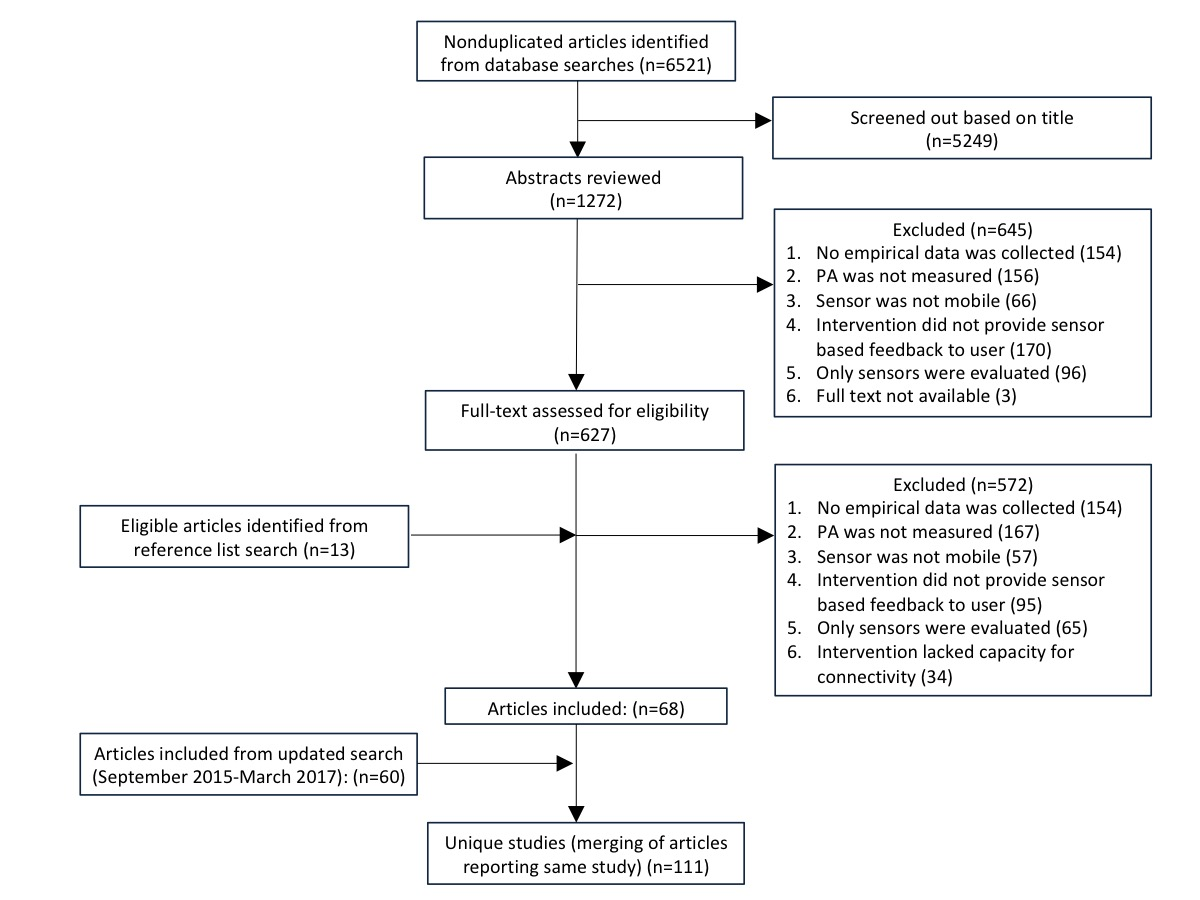
Preferred Reporting Items for Systematic Reviews and Meta-Analyses (PRISMA) diagram.

A total of 6521 articles were retrieved during the initial database search (see Figure 1). After title screening, we reviewed 1272 abstracts and excluded 645 articles that did not meet the inclusion criteria. The full texts of the remaining 627 articles, and an additional 13 articles identified from reference lists searches, were read. Furthermore, 572 studies were excluded, leaving 68 articles. An additional 60 articles were included from the updated search in March 2017 (where we reviewed 557 abstracts and excluded 338 articles that did not meet criteria; then 219 full texts and excluded 159 articles that did not meet criteria). Overall, from the 1829 abstracts and 858 full texts read, a total of 128 articles were included in the review [39-166], representing 111 unique studies.

### Study Characteristics

The study characteristics are presented in Multimedia Appendix 1. Of the included studies, 22/111 (19.8%,) were protocols. Over half (55.0%, 61/111) were published in 2015 or later. Many (42.3%, 47/111) were conducted in the United States. The majority of studies (93.0%, 103/111 ) involved adult participants; 8/111 studies (7.0%) involved children and adolescents. Participant numbers ranged from 2 [39] to 2980 [40]: 18.9% (21/111) of studies contained fewer than 13 participants. Study duration ranged from less than a day to 52 weeks. Intervention characteristics are included in Multimedia Appendix 2.

### Research Designs

Of the included studies (see Multimedia Appendix 3), 61/111 (55.0%) used an RCT design. Most of these (66%, 40/61) were 2-group RCTs; 12 (23%, 12/61) were 3-group RCTs and 9 (15%, 9/61) were 4-group RCTs. Control group participants within RCTs received (1) standard care or minimal contact or print materials (39%, 24/61); (2) active comparison treatments (26%, 16/61); (3) noninteractive devices that did not display feedback (18%, 11/61); or (4) waitlist or no intervention (16%, 10/61). The remaining studies included 23/111 (22.5%) repeated measures designs; 11/111 (9.9%) nonrandomized group designs; 10/111 (9.0%) case studies (6/10 [60%] of which included an experimental baseline phase) and 4/111 (3.6%) observational studies. Only 2/111 studies (1.8%) used rapid research designs: one single-case design and one MOST.

As shown in Textbox 2, studies investigated a variety of intervention components, including the addition of apps or wearables to non-technology based interventions delivered by health care professionals, and a range of in-app components, such as automated adaptive goal-setting versus static or manual input of goals, and different social components.

### Objectives and Data Collection Methods

Multimedia Appendix 3 shows the objectives that each study investigated effectiveness, engagement, acceptability and/or usability. Almost all studies (96.4%, 107/111) investigated effectiveness, including 14/111 (12.6%) that explored preliminary impact using only descriptive statistics or visual analysis. Only 35/111 studies (31.5%) investigated effectiveness, engagement and acceptability together, and 14 of these (40%, 14/35), did not use inferential statistics analysis to assess effectiveness. Usability was assessed in 16/111 studies (14.4%).

### Effectiveness

The majority of studies (90.9%, 101/111) used sensors to measure physical activity. These were most often the in-device sensors used to deliver feedback on physical activity (67.6%, 75/111) (eg, Fitbit [105,162]). Some studies used external sensors (eg, Acti-Graph GT3X [ActiGraph, Shalimar, FL, USA], Sensewear Armband [BodyMedia, Inc., Pittsburgh, PA], Omron pedometer [Omron Healthcare, Inc., Bannockburn, I]), instead of, or in triangulation with, in-device sensors (23.4%, 26/111). Physical activity data collected via in-device and external sensors included step counts (eg,[159]) and time spent being active (eg, [84,151]). An external device was significantly more likely to be used in RCTs than in other research designs (χ^2^_1_=7.8, *P*=.005).

Of the included studies, 10/111 (9.0%) used a questionnaire alone to measure self-reported physical activity, and 17/111 (15.0%) used a questionnaire to triangulate with sensor data. Questionnaires included the International Physical Activity Questionnaire [167], the Community Health Activities Model Program for Seniors [168], the Recent Physical Activity Questionnaire [169], the Godin Leisure-Time Exercise Questionnaire [170], the Active Australia survey [171], the 7-day Sedentary and Light Intensity Physical Activity Log (7-day SLIPA Log [172], the Yale Physical Activity Scale [173], and the WHO Global Physical Activity Questionnaire [174].

### Engagement

Engagement (ie, users’ interaction with the device and usage behavior) was measured by 58/111 studies (52.3%) (Multimedia Appendix 3), with most (91%, 53/58) using device-generated logs to do so. Seven (12%, 7/58) used both logs and self-report questionnaires as a form of triangulation, and 5/58 (8%) used self-report questionnaires alone. Three dimensions of engagement were identified (1) frequency or amount of use; (2) depth of engagement (ie, active vs passive); and (3) length of use. These are described in Textbox 3.

**Intervention components and features investigated for impact on physical activity in included studies.**

- Addition of apps and wearables to nontechnology based interventions with health care professionals [122,133,137].
- Addition of gamification features [115,118,123,148], financial incentives [57,119,144,152,154] and notifications or short messaging service (SMS) texts [102] to self-monitoring interventions.
- Automation of self-monitoring and goal-setting, including automated activity recognition versus manual input by the user [54,73] and automated adaptive goal-setting versus standard static or manual input of goals [50,124,127,150].
- Different social app features that support cooperation or competition [164] or accountability [161], social gaming and interaction [114], and personal versus group-based feedback [92,153]
- Different types of feedback messages, including positive or negative [99] and novel versus familiar [124].
- Different prompt frequencies [104].

**Dimensions of engagement assessed by included studies.**

Frequency or amount of use

- Number of log-ins [83,137], number of times app opened [92,103], number of days device worn [139,165,166], self-reported frequency of viewing activity trackers [136]
- Use of social features, including self-reported frequency of viewing social media messages [139], number of social media messages sent [50,106,130,140], number of times leader board page accessed [139], number of likes or posts on Facebook [61], number of YouTube video views [160]
- Frequency of use by health care professional [52]
- Number of physical activity uploads [137]
- Amount of present or missing sensor data [156]

Depth of engagement (ie, active vs passive)

- Whether or not the user manually adjusted preset goals [116,124,150] or the physical activity levels that were inferred by the device [54]
- Number of missions or challenges completed [61]
- Logs indicate glancing (5-second intervals with no looking back at step history), review (use or interaction of up to 60 seconds, scrolling through step history), and engagement (use or interaction over 60 seconds, scrolling through step history), and also time between periods of engagement [124]

Length of use

- Number of times app opened across weeks [92], number of users continuing to post to community board [139], and number of days app used post study [97]

### Acceptability

Of the studies included, 64/111 (57.5%) investigated acceptability (ie, users’ subjective perceptions and experiences; see Multimedia Appendix 3). Most used questionnaires (64%, 41/64), and just over half (53%, 34/64) used qualitative interviews or focus groups, either alone or in addition to questionnaires. Questionnaires included a range of standardized questionnaires (eg, the IBM Computer Usability Satisfaction Questionnaire [175], the Persuasive Technology Acceptance Model Questionnaire [176], the Intrinsic Motivation Inventory [177], the Fun Toolkit [178] and the Working Alliance Inventory [179]), or questionnaires developed especially for the study (eg, [73,88]). A few studies employed user logs (11%, 7/64), of which, 3 used device-generated usage logs as a “proxy” of users’ interest [135] or preferences [143,150]; 4 used user-entered text (eg, the content of social media messages to understand the types of social support that users experienced [86,106,130], and digital diary entries to understand experiences of using the device [106,127]). Studies that used text-based logs also employed face-to-face qualitative methods (ie, interviews, focus groups) or questionnaires, in addition to collecting log data. Five dimensions were identified in relation to measuring acceptability (1) appreciation; (2) perceived effectiveness and usefulness; (3) user satisfaction; (4) users’ intention to continue use of the app or device, and (5) social acceptability. These are described in Textbox 4.

### Usability

Usability was investigated by 16 studies (14.4%, 16/111), out of which, 9 (56%, 9/16) used questionnaires (eg, the System Usability Scale [180]); 4 (25%, 4/16) used interviews; 2 (13%, 2/16) used focus groups; and 1 (6%, 1/16) [70] used observation of participants’ completing timed tasks. Three dimensions were identified in relation to assessing usability (1) burden of device wear and use, (2) interface complexity, and (3) perceived technical performance. These are described in Textbox 5.

**Dimensions of acceptability assessed by included studies.**

Appreciation

- Appreciation or liking of the app [39,126,141]
- Whether the app or wearable was perceived as enjoyable, fun, entertaining [61,74,123,127]
- Whether the app or wearable was perceived as pleasant [103], attractive or visually appealing [160]
- What was “missed” about a feature once withdrawn [128]
- How the user “felt” about the app or wearable and its components [39,68,79,99]
- Users’ interest and preferences [135,143]
- Teachers’ perceptions of whether the app or wearable appealed to students [61]
- Self-reported motivation to pay attention [127]
- Trustworthiness of the app or wearable [39,73]
- Perceived advantages and disadvantages of using the app or wearable [53,115]

Perceived effectiveness and usefulness

- Users’ views on whether the app or wearable increased, or will continue to increase and promote, physical activity [39,52,70,75,79,94,103,113,122,123,126,143,145]
- Practice nurses’ perceptions of effectiveness for patients [132]
- Users’ perceived usefulness or helpfulness of the app or wearable [39,74,103,116] and its components [52,59,136,165,166] in self-monitoring [54], supporting fitness and physical activity [118,136], and supporting them to stay motivated [163]
- Users’ perceived persuasiveness or helpfulness of the app or wearable in achieving goals [160]
- Ability of the app or wearable to provide answers to health-related questions [160] and insight into physical activity or health conditions [52]
- Health care professionals’ perceptions of the usefulness of information about patients’ physical activity or health condition and whether it supported engagement with patients’ home care [52]

Satisfaction

- General user satisfaction [41,75]
- User satisfaction with number of reminder short messaging service or calls received [136]
- User satisfaction with length of intervention [61,160]
- User satisfaction with level of personalization [127] and feedback provided by the app or wearable [54]
- Likelihood of users recommending the app or wearable to a friend or other people [116,139,162,165,166]
- Satisfaction with different components or features [116,122,129,145,163,165,166]
- Likelihood of physicians recommending the app or wearable to patients [55]

Users’ intention to continue use of the app or wearable

- Intention or willingness to use after the study [39,92,97,103]
- Intention to continue if user had to pay for the app or wearable [156], or intention to purchase the app or wearable after the study [139,160]
- How regularly the user intended to use the app or wearable after the study [54,55]

Social acceptability

- Whether the app or wearable was noticed and remarked upon by others [79,128] or prompted discussion with others [52].
- Whether the app or wearable was used by important others [39].
- Users’ attitudes towards sharing data with other people [130].
- Social encouragement [123] and social support received when using (including via) the app or wearable [85,86,110]
- Level of social bonding between the user and virtual coach [73]
- Users’ preferences in using individual versus social features [161]
- Whether notifications were received at a socially acceptable time and place [147] or interfered with users’ daily activities [122]

**Dimensions of usability assessed by included studies.**

Burden of wear and use

- Ease of wear [145], burden or restriction in wearing the device, physical discomfort [142,159], usability regarding the device size [81], suggestions for alternative wear locations [116]
- Ease of use [39,49,143] when syncing to Web-based databases [142,159] and when charging the device [81]
- Whether device interfered with daily activities [122]

Interface complexity

- Complexity and intuitiveness [65], accessibility [159], and comprehension of physical activity feedback [160]
- Ease of reading information [122]
- Difficulties using the interactive interface, users’ speed when completing in-app tasks [70]

Perceived technical performance

- Users’ perceptions of the accuracy of the app or wearable in recognizing or inferring their physical activity [54,65,142]
- Technical difficulties or barriers encountered by users [113,116]

## Discussion

### Principal Findings

Of the 111 studies included, around half were published between 2015 and 2017, 55.0% were RCTs, and only 2 studies used rapid designs. Almost all studies measured physical activity objectively using sensors (either in-device or external), with RCTs more likely to employ external sensors (accelerometers). Less than one-third of the studies investigated effectiveness, engagement, and acceptability together. According to our working definitions, studies that measured engagement mostly used device-generated logs to assess the frequency, depth, and length of engagement. Studies exploring acceptability most often used questionnaires and/or qualitative methods to assess appreciation, perceived effectiveness and usefulness, satisfaction, users’ intention to continue use of the app or device, and social acceptability. A small number of studies explored usability of the device (including burden of sensor wear and use, interface complexity, perceived technical performance) using questionnaires, qualitative methods, or participant observation.

The fact that more than half of the included studies were published between 2015 and 2017 demonstrates that research into the impact of physical activity apps and wearables is a growing area of interest, underscoring the timeliness of this review. Despite this, we found that only 2 studies used the rapid research designs that have been recommended for evaluating mHealth technologies (single-case design [131] and the MOST approach [164]). A low uptake of rapid research designs was similarly reported in a recent review of clinical mHealth app evaluations [34]; however, while the vast majority of evaluations of clinical apps were RCTs, our findings show that evaluations of physical activity apps and wearables use alternative research designs (including repeated measures designs, nonrandomized group designs, case studies and observational studies) more often. This may reflect the interdisciplinary nature of our review, and the view held by some HCI researchers that RCTs, as well as being impractical and resource intensive, are of limited usefulness [181]. It is nevertheless surprising that few studies used single-case designs and new factorial approaches, as it has been suggested that mHealth technologies can support the data collection procedures and experimental setup these research designs require (ie, frequent measurement and several experimental conditions) [25,26,182].

Further research is needed to explore the reasons that rapid research designs are not being used. It could be that the requirements for these designs are not feasible for some research projects. MOST, for example, requires several decisions to be made in advance of conducting the trial (eg, deciding which specific theory-based components of the intervention should be tested, and assessing the feasibility of carrying out a research design that can often require large sample sizes [29]). These requirements can themselves be time and resource intensive [183]. Barriers to using rapid research designs may also be conceptual: preliminary evidence suggests that the value of, and requirements for, single-case designs were not fully understood by clinical health practitioners [184], which may also apply to mHealth researchers.

In addition to effectiveness, assessing user engagement and acceptability are important to (1) generate a better understanding of the overall impact; (2) explain variation in the outcomes; and (3) reveal (potentially interactive) influences on effectiveness [16,19]. Despite this, only around one-third of the studies (32.0%) investigated all 3 objectives together. Furthermore, 40.0% of these did not use inferential statistics to assess effectiveness (instead using descriptive statistics and visual analysis), and almost one-fifth of all studies (18.9%) contained fewer than 13 participants. These preliminary, small-N studies are typical of iterative HCI research focused on developing novel technologies [185], and are unlikely to be sufficiently powered to test important hypotheses on mediators of effectiveness [17,186]. Although this study did not explore the specific statistical analyses undertaken, Bayesian methods are considered a promising approach for mHealth evaluations [13,25,187] and can be used to investigate mediating variables in small-N studies [188]. As such, Bayesian methods could be key when exploring results from early developmental evaluations to reveal potential relationships between mHealth engagement, acceptability, and effectiveness.

Many evaluations of physical activity apps and wearables appear to be taking advantage of efficient data collection methods: two-thirds of studies employed in-device sensors in smartphones and wearables to measure physical activity. The fact that RCTs used external, validated sensors more often than other study designs exacerbates their inefficiency (eg, through adding extra resource costs [189]). Furthermore, using external sensors often involves measurement procedures that may reduce the generalizability of findings to real-world contexts (eg, requiring participants to wear additional devices and visit the lab). The coupling of gold standard RCTs and sensors with established validity indicates a well-grounded concern for methodological rigor. Yet, balancing this need for rigor with the need for efficiency requires further investigation. Addressing any “trade-offs” between efficiency and rigor when evaluating physical activity apps and wearables (and mHealth technologies more generally [11]) will require, at the very least, understanding the validity and reliability of internal sensors. Evidence could be quickly accumulated using industry-based “research libraries,” such as Fitabase [190], and then used to inform decision making when designing a pragmatic evaluation. Relatedly, empirical evidence is needed to support recently proposed digital health evaluation models that outline all phases of the research process [191,192]: these frameworks combine HCI and implementation science methods to ensure evaluations are both rigorous and sustainable in real-world settings.

Most studies that measured engagement, used device-generated logs: these can be more efficient than qualitative self-report methods, which can be time-consuming and burdensome [20]. In contrast, acceptability was generally assessed via questionnaires and/or qualitative face-to-face methods. HCI researchers have emphasized the need to collect subjective qualitative data alongside device-generated logs to fully understand not only “what” people are doing but “why” [32,33]. We found a handful of studies (11%) used log data (eg, device-generated usage logs or user-entered text logs) to assess some dimensions of acceptability. The validity of this approach (ie, whether either form of log data can sufficiently capture the rich contextual details typically afforded by traditional qualitative methods) should be explored. For example, device-generated logs showing continued engagement with the app could imply user “satisfaction,” “appreciation,” and “perceived effectiveness or usefulness of the app,” whereas investigating “social acceptability” (eg, user attitudes toward publicly sharing data) may require user-entered text logs (eg, from digital diaries, Web-based questionnaires, and social media posts), or even face-to-face methods.

In this review, we defined engagement as users’ interaction and usage behavior [21,22] and acceptability as users’ subjective perceptions and experiences. The dimensions of engagement and acceptability that we identified rested upon these working definitions. There is still no consensus in mHealth and related fields on what constitutes engagement and acceptability, and how each should be measured. One recent review [23] proposed that engagement is a multidimensional construct that includes not only dimensions related to “usage” (ie, amount, frequency, depth, and duration of engagement) but also subjective experiences of engagement (eg, affect, attention, and interest). Another review conceptualized engagement as “any process by which patients and the public became aware of or understood a digital health intervention” [193]. In response to varying definitions of engagement, researchers have undertaken valuable consensus-building exercises (and have emphasized the need to focus on “effective engagement” that accounts for engagement with behavior change) [20]. Clarification and consensus will advance our understanding of how engagement and acceptability may individually, or interactively, influence effectiveness.

A few studies assessed *usability.* In line with other conceptualizations of usability (ie, whether the device or app is easily used to achieve specified goals successfully and quickly [194,195]), we distinguished usability from acceptability by considering it to be a characteristic of the device. Understanding the degree to which usability varies across users and interacts with context to ultimately influence effectiveness (as opposed to being a stable device characteristic) will determine whether it should be assessed during within effectiveness evaluations (or instead optimized beforehand).

The screening process in this interdisciplinary review involved a very high number of abstracts and full papers being read to identify the final studies for inclusion. Many of the articles retrieved from the database searches had ambiguous titles; and many authors omitted key study details from their abstracts. Furthermore, data extraction from the full-text articles involved negotiating different publication formats across disciplines. These challenges meant the review process was far more time-consuming than originally envisaged. Currently, HCI studies are not required to follow heath science reporting guidelines that promote the inclusion of specific study details in titles and abstracts [196]. Standardized reporting drawing on existing guidelines (eg, CONSORT-EHEALTH [197]) would allow different disciplines to more easily synthesize the large amount of research that is being conducted in this area and would also aid current efforts to develop automated processes to increase the accessibility of evidence from digital health publications [198].

### Limitations

The review was conducted systematically and comprehensively across health, clinical, and computing science databases. However, the scoping methodology followed did not include any assessment of the methodological quality of studies [37]. The focus on physical activity, engagement, and acceptability (and usability) meant that other important aspects of evaluation, such as reach and uptake, secondary clinical and psychological outcomes, cost-effectiveness, and the statistical analysis methods that studies used, were not reported. Furthermore, without established definitions of engagement and acceptability, the dimensions identified in this review are necessarily provisional.

The review did not examine the context in which apps and wearables were developed and evaluated, such as within academia versus industry. The development context may influence the assessment and reporting of engagement, acceptability, usability, and effectiveness of the apps and wearables. Commercially-developed apps, for example, often do not incorporate behavior change techniques that improve effectiveness [199-202] and may focus more on enhancing user experience: therefore, industry professionals may be more likely to assess engagement, acceptability, and usability rather than effectiveness. Finally, to understand whether studies employed in-device sensors to measure physical activity, studies were included only if they evaluated apps and wearables that provide sensor-based feedback on physical activity. Therefore, the findings of the review cannot be generalized to other technologies or health behaviors.

### Future Research

Future research should investigate why recommended rapid research designs are not yet widely adopted. For example, qualitative explorations of researchers’ and industry professionals’ perceptions and daily research practices and experiences would allow an understanding of the practical challenges in using rapid designs in academia and industry; and feasibility studies should explore the extent to which rapid designs can be supported and automated by mHealth technologies [11]. Consensus is needed on how to define and distinguish engagement and acceptability, and on the specific dimensions of these constructs, which could then be tested as potential mediators and moderators of effectiveness. Finally, the validity and usefulness of logging methods for assessing acceptability should be explored.

### Conclusions

Despite the rapid increase of evaluations of the impact of physical activity apps and wearables, few are optimized in relation to efficiency and assessment of the key constructs of effectiveness, engagement, and acceptability. The findings of this review will inform future guidance to support health and HCI researchers in making greater use of rapid research designs (eg, single-case designs), in-device sensors, and user-logs to collect effectiveness, engagement, and acceptability data. The difficulties encountered in conducting this interdisciplinary review also highlight the need for standardized reporting guidelines. These would facilitate the synthesis of evidence across health and HCI disciplines, and thus support rapid advancement in understandings of the extent to which apps and wearables can support users to become more physically active.

## Appendix

Multimedia Appendix 1 Study characteristics.

Multimedia Appendix 2 Intervention characteristics.

Multimedia Appendix 3 Research designs used in included studies and objectives investigated.

## Abbreviations

ACM: Association for Computing Machinery Digital Library
HCI: human computer interaction
IEEE: Institute of Electrical and Electronics Engineers
mHealth: mobile health
MOST: multiphase optimisation strategy
RCTs: randomized controlled trials
