# Supplementary material for: Evaluating the Impact of Physical Activity Apps and Wearables: Interdisciplinary Review
Source: JMIR Mhealth Uhealth. 2018 Mar 23;6(3):e58. doi: 10.2196/mhealth.9054 (PMC5889496; doi:10.2196/mhealth.9054)
Supplement: Multimedia Appendix 2 [file mhealth_v6i3e58_app2.pdf]

Multimedia Appendix 2: Intervention characteristics

Study	Technologies	Key intervention features[a,b]
Walters et al 2010 [41]	Smartphone app + web + SMS	Motivational messages, goal-setting with HCP, counselling with HCP
Kharrazi et al 2011 [42]	Wireless pedometer + web	Interactive personal health record, social network, automated goal-setting, reward badges
Pellegrini et al 2012 [43]	Smartphone app	Group sessions, coaching calls, goal-setting for weight, diet and PA, monetary rewards
Jimenez Garcia et al 2013 [44]	Smartphone app	Visualised feedback and goal-setting, vibration prompts, mood diary
Geraedts et al 2014 [45]	Tablet	Exercise videos and telephone coaching
Recio-Rodriguez et al 2014 [46]	Smartphone app	Automated advice, additional manual input of PA
Clayton et al 2015 [47]	Wearable + web	Share activity profile with HCP, goal-setting with HCP
Cooper et al 2015 [48]	Wearable + wireless scales + web	Online detailed FB, goal-setting for weight loss, online virtual coaching
Granado-Font et al 2015 [49]	Smartphone app	Diet monitoring, Social network
Hurley et al 2015 [50]	Wearable + SMS	Adaptive or static goal-setting, immediate or delayed monetary incentives
Pellegrini et al 2015 [51]	Smartphone app	Diary, goal-setting for diet, exercise and sedentary time
Agboola et al 2016 [52]	Smartphone app + wearable	Tailored messaging, community feature for social support and comparison, goal-setting, educational library, portal for providers
Amorim et al 2016 [53]	Smartphone app + wearable	Face-to-face coaching with HCP, goal-setting with HCP, personalised messages
Duncan et al 2016 [54]	Smartphone app + wearable	Educational materials (guidelines, strategies to promote change in behaviour), goal setting with detailed visual feedback
Jones et al 2016 [55]	Smartphone app + wearable	Motivational messages and self-tracking of daily pain and mood
Ortiz et al 2016 [56]	Wearable	Feedback on PA
Shin et al 2016 [57]	Smartphone app + wearable	Feedback on PA, financial incentives
Taylor et al 2016[58]	Smartphone app	Feedback on PA
van Nassau et al 2016 [59]	Smartphone app + wearable + web	Feedback on PA and sedentary time, social networking, social game, face-to-face classroom discussions and graded group-based PA led by community coaches
Brickwood et al 2017 [60]	Smartphone app + wearable	Health tips and remote feedback from HCPs, telephone counselling
Ridgers et al 2017 [61]	Smartphone app + wearable + web	Interactive weekly individual and/or team missions or challenges, infographics, motivational videos and social

		forums
Wolk et al 2017 [62]	Wearable	Feedback on PA
Slootmaker et al 2005 [63, 64]	Wearable + web	Online exercise planning and goal-setting
Fujiki et al 2007 [65]	PDA	Scheduling, automated advice, forum, reminders
Hurling et al 2007 [66]	PDA	Avatar, competitive games, game status notifications
Polzien et al 2007 [67]	Wearable + web	Feedback on PA, calories and weight, online self-monitoring, Goal-setting with HCP
Consolvo et al 2008 [68]	Smartphone app	Mobile wallpaper supports continuous graphical feedback on PA, goal-setting, diary, manually edit detected PA
Faridi et al 2008 [69]	Wireless pedometer	Reminders, share data with HCP
Fujiki et al 2008 [70]	Pda	Avatar, competitive games, game status notifications
Lacroix et al 2008 [71, 72]	Wearable + web	LED lighting provides feedback, online detailed feedback, goal-setting
Albaina et al 2009 [39]	Wearable + interactive photo frame	Graphical feedback, automated goal-setting, virtual coach
Bickmore et al 2009 [73]	PDA + web	Virtual, context-aware coach
Fialho et al 2009 [74]	Wearable + web	Social networks, messaging, accept challenges, user can comment on PA, virtual coach
Arsand et al 2010 [75]	Smartphone app	Automated goal-setting, health information access
Mattila et al 2010 [76,77]	Smartphone app + wireless scales	Diary, graphical feedback
Penados et al 2010 [78]	Interactive pocket toy	Mood of toy provides feedback
Lim et al 2011 [79]	Wearable	LED light intensity provides feedback
Shuger et al 2011 [80]	Wearable + web	Feedback on PA, telephone counselling with HCP
Burns et al 2012 [81]	Wearable + web	Wearable LED flashes/changes colour if others physically active
Gomes et al 2012 [40]	Wearable + web	Online game, monetary rewards, friend list, newsfeed
Pellegrini et al 2012 [82]	Wearable + web	Feedback on PA, calories, and weight, online detailed feedback, and goal-setting with HCP
Reijonsaari et al 2012 [83]	Wearable + web	Online detailed feedback, and telephone/online counselling with HCP
Van Hoye et al 2012 [84]	Wearable	Feedback on calories and PA, goal-setting, and HCP coaching sessions
Xu, Poole, et al 2012 [85,86]	Wireless pedometer + web	Online team game, avatars, message board, teacher customisation

Barwais et al 2013 [87]	Wearable	LED lighting provides feedback, online detailed feedback, goal-setting and motivational messages
Bentley et al 2013 [88]	Smartphone app + Web	Widget shows statistical relationships between diet, PA, and contexts via context-sensing
Chatterjee et al 2013 [89,90]	Smartphone app + SMS	Motivational messages, personalised newsletter
Fitzsimmons et al 2013 [91]	Wearable	Supports consultations and goal-setting with HCP
Harries et al 2013 [92,93]	Smartphone app	Avatar, social comparison messages
Hirano et al 2013 [94]	Smartphone app	Wallpaper supports continuous graphical FB, vibration prompts, goal-setting and scheduling
Khalil & Abdallah 2013 [95]	Smartphone app	Social networks, share PA data with team, view team progress
Khan & Lee 2013 [96]	Smartphone app + SMS	Automated advice based on activity levels and location detected
King et al 2013 [97] [98]	Smartphone app	Wallpaper for continious PA feedback, goal-setting, automated "just-in-time" advice, message boards, avatar
Nakajima et al 2013 [99]	Wireless pedometer + interactive photo frame	Interactive painting provides feedback on PA
Tabak et al 2013 [100,101]	Smartphone app + web + SMS	Virtual coach, automated advice, social graphs, diary
Valentin & Howard 2013 [102]	Smartphone app	Vibration prompts, avatar, goal-setting
Bond et al 2014 [103,104]	Smartphone app	Just-in-time audio reminder for PA break, graphical feedback
Caulfield et al 2014 [105]	Wearable + web	Online detailed feedback
Chen & Pu 2014 [106]	Smartphone app	Dyads/friends share badges, different social settings
Glynn et al 2014 [107-109]	Smartphone app	Graphical feedback, goal-setting
Miller et al 2014 [110]	Wireless pedometer + web	Online team game, message board
Thompson et al 2014 [111]	Wearable + web	Online detailed feedback, online PA education, GS, counselling with HCP
Thorndike et al 2014 [112]	Wearable + web	Online detailed FB, monetary rewards, team-based competition
Verwey et al 2014 [113]	Smartphone app + Web	User can enter comments about PA, goal-setting with HCP
Walsh et al 2014 [114]	Wearable + web	User profile, social network, currency based game
Zuckerman et al 2014 [115]	Smartphone app	Wallpaper supports continuous graphical feedback, avatar, automated goal-setting, notifications
Cadmus-Bertram et al 2015 [116,117]	Wearable + web	Self-regulation skills (goal setting and frequent behavioural feedback)
Direito et al 2015 [118]	Smartphone app	Education, social networking/forums/messaging

Finkelstein et al 2015 [119,120]	Wearable + web	Monetary rewards, charity donation
Frederix et al 2015 [121]	Wearable + SMS	Goal setting, weekly advice, and exercise training with HCP
Frederix et al 2015 [122]	Smartphone app + wearable + SMS	Tailored exercise training protocols, encouraging messages that change over time texts regarding diet and smoking
Garde et al 2015 [123]	Smartphone app	Team game, reward badges
Gouveia et al 2015 [124]	Smartphone app	Goal setting, contextualizing physical activity via context-sensing, and textual feedback that continuously updates
Guthrie et al 2015 [125]	Wearable + web	Goal-setting, rewards (badges, monetary incentives), avatar, social comparison, social facilitation
Komninos et al 2015 [126]	Smartphone app	Audio feedback via degraded music quality until user reaches target cadence
Lee et al 2015 [127]	Wearable + web	Personalised planning and goal-setting
Lee et al 2015 [128]	Wearable	Material engraver; tailored feedback using representative patterns
Martin et al 2015 [129]	Smartphone app + wearable + SMS	Automated coaching texts with positive re-enforcement messages
Munson et al 2015 [130]	Wearable + web	Custom website which links to wearable and social networks, automated goal-setting, public goal commitments
Rabbi et al 2015 [131]	Smartphone app	Automated and personalised suggestions and feedback on diet and PA using recommender systems
Verwey et al 2015 [132-134]	Smartphone app + Web	Annotate and comment osn daily PA, share with HCP and goal-setting
Wadwha et al 2015 [135]	Smartphone app + web	Notifications to increase activity time, leadership board, monetary rewards
Wang et al 2015 [136]	Smartphone app + wearable + SMS	Activity prompt and goal-setting via SMS
Watson et al 2015 [137]	Wearable + wireless scales + web	Goal-setting and automated feedback on weight, diet and PA, meal suggestions, social support via community forum
Broekhuizen 2016 [138]	Wearable + web + SMS/email	Goal-setting, sustainability support, personal e-coach sends PA advice
Butryn et al 2016 [139]	Wearable + web	Goal-setting, leaderboard to support social comparison, social support
Choi et al 2016 [140]	Smartphone app + wearable	Daily text or video message, interactive automated feedback and advice, images and video clips regarding posture and stretching
Ciman et al 2016 [141]	Smartphone app	Stair-climbing game, with individual, collaborative and competitive modes
Darvall et al 2016 [142]	Wearable	Feedback on PA
Ding et al 2016 [143]	Smartphone app + wearable	Just-in-time context-aware reminders with explanations of context to support behaviour change
Fennell et al 2016 [144]	Wearable	Positive reinforcement through material incentives, negative reinforcement via monetary buy ins.

Garde et al 2016 [145]	Smartphone app + wearable	PA is rewarded by playtime and game incentives such as special features, unlocked levels, competitive score keeping, and peer interaction
Gilson et al 2016 [146]	Smartphone app + wearable	Feedback on PA, dietary logging
Glance et al 2016 [147]	Wearable + web	Team averages, reward badges, prizes, Leaderboards, Social Network
H-Jennings et al 2016 [148]	Wearable + web	Leaderboard to track individual daily progress, a forum for discussions, references on PA and sleep
Hartman et al 2016 [149]	Smartphone app + wearable + web	Self-regulatory skill building, goal-setting
Herrmany et al 2016 [150]	Smartphone app	Automated goal recommendations, manual goal setting, record reference routes
Melton et al 2016 [151]	Smartphone app + wearable + web	Weekly health tips and reminders
Patel et al 2016 [152-154]	Smartphone app + wearable + SMS	Individual or combined financial incentives, different social comparison settings
Paul et al 2016 [155]	Smartphone app	Avatar, view when others' active, individual goal-setting, image-based rewards
Quintiliani et al 2016 [156]	Wearable + wireless scale + telephone	Telephone counselling with HCP
Vorrink et al 2016 [157]	Smartphone app + web + SMS	Website for physiotherapists to monitor patients and adjust goals
Walsh et al 2016 [158]	Smartphone app	Goal-setting, visually appealing graphic display of step-count history
Yingling et al 2016 [159]	Wearable + web	Online diary
Ashton et al 2017 [160]	Smartphone app + wearable + web	Educational website, social network
Chen et al 2017 [161]	Smartphone app + wearable	Team dyads, leaderboard and collective goal setting for social cooperation and comparison
Chung et al 2017 [162]	Smartphone app + wearable	Graphical feedback on PA, social network
Gell et al 2017 [163]	Wearable + SMS	Location-based feedback including a map of MVPA bout locations, discussion, goal-setting and planning with HCP
McMahon et al 2017 [164]	Wearable	Feedback on PA, small in-person group discussions to support interpersonal (social support, social comparison) and intrapersonal (goal-setting) behaviour change strategies
Neil-Sztramko et al 2017 [165]	Wearable + web	Feedback on PA, remote behavioural counselling sessions with HCP
Valle et al 2017 [166]	Wearable + wireless scales + web	Weight gain education, real-time feedback and weekly tailored feedback

a All interventions included sensor-based feedback on physical activity
b PA = Physical activity, HCP = Healthcare Professionals

References

39. Albaina I, Visser T, van DMC, Vastenburg M. Flowie: A persuasive virtual coach to motivate elderly individuals to walk. 2009. Presented at: 3rd International Conference on Pervasive Computing Technologies for Healthcare (PervasiveHealth) 2009; Apr 1-3; London.[CrossRef]
40. Gomes N, Merugu D, O'Brien G, Mandayam C, Jia SY, Atikoglu B. Steptacular: An incentive mechanism for promoting wellness. 2012. Presented at: 4th International Conference on Communication Systems and Networks (COMSNETS) 2012; Jan 3-7; Bangalore.[CrossRef]
41. Walters DL, Sarela A, Fairfull A, Neighbour K, Cowen C, Stephens B, Sellwood T, Sellwood B, Steer M, Aust M, Francis R, Lee C, Hoffman S, Brealey G, Karunanithi M. A mobile phone-based care model for outpatient cardiac rehabilitation: the care assessment platform (CAP). BMC Cardiovasc Disord 2010; 10:5[FREE Full text][Medline][CrossRef]
42. Kharrazi H, Vincz L. Increasing physical activity by implementing a behavioral change intervention using pervasive personal health record system: an exploratory study. 2011. Presented at: 6th International Conference on Universal Access in Human-Computer Interaction (UAHCI). HCI International 2011; Jul 9-14; Orlando, FL.[CrossRef]
43. Pellegrini CA, Duncan JM, Moller AC, Buscemi J, Sularz A, DeMott A, Pictor A, Pagoto S, Siddique J, Spring B. A smartphone-supported weight loss program: design of the ENGAGED randomized controlled trial. BMC Public Health 2012; 12:1041[FREE Full text][Medline][CrossRef]
44. Jimenez GJ, Romero N, Keyson D, Havinga P, editors. ESTHER 1.3: integrating in-situ prompts to trigger self-reflection of physical activity in knowledge workers. 2013. Presented at: ChileCHI'13 Chilean Conference on Human-Computer Interaction 2013; Nov 11-15; Temuco, Chile.[CrossRef]
45. Geraedts HAE, Zijlstra W, Zhang W, Bulstra S, Stevens M. Adherence to and effectiveness of an individually tailored home-based exercise program for frail older adults, driven by mobility monitoring: design of a prospective cohort study. BMC Public Health 2014; 14:570[FREE Full text][Medline][CrossRef]
46. Recio-Rodríguez JI, Martín-Cantera C, González-Viejo N, Gómez-Arranz A, Arietaleanizbeascoa MS, Schmolling-Guinovart Y, Maderuelo-Fernandez JA, Pérez-Arechaederra D, Rodriguez-Sanchez E, Gómez-Marcos MA, García-Ortiz L. Effectiveness of a smartphone application for improving healthy lifestyles, a randomized clinical trial (EVIDENT II): study protocol. BMC Public Health 2014; 14:254[FREE Full text][Medline][CrossRef]
47. Clayton C, Feehan L, Goldsmith CH, Miller WC, Grewal N, Ye J, Yoo JY, Li LC. Feasibility and preliminary efficacy of a physical activity counseling intervention using Fitbit in people with knee osteoarthritis: the TRACK-OA study protocol. Pilot Feasibility Stud 2015 Aug; 1:30[FREE Full text][Medline][CrossRef]
48. Cooper Andrew J M, Dearnley Katie, Williams Kate M, Sharp Stephen J, van Sluijs Esther M F, Brage Soren, Sutton Stephen, Griffin Simon J. Protocol for Get Moving: a randomised controlled trial to assess the effectiveness of three minimal contact interventions to promote fitness and physical activity in working adults. BMC Public Health 2015 Mar 27; 15:296[FREE Full text][Medline][CrossRef]
49. Granado-Font E, Flores-Mateo G, Sorlí-Aguilar M, Montaña-Carreras X, Ferre-Grau C, Barrera-Uriarte M, Oriol-Colominas E, Rey-Reñones C, Caules I, Satué-Gracia E. Effectiveness of a Smartphone application and wearable device for weight loss in overweight or obese primary care patients: protocol for a randomised controlled trial. BMC Public Health 2015 Jun 04; 15:531[FREE Full text][Medline][CrossRef]
50. Hurley JC, Hollingshead KE, Todd M, Jarrett CL, Tucker WJ, Angadi SS, Adams MA. The Walking Interventions Through Texting (WalkIT) Trial: Rationale, Design, and Protocol for a Factorial Randomized Controlled Trial of Adaptive Interventions for Overweight and Obese, Inactive Adults. JMIR Res Protoc 2015 Sept 11; 4(3):e108[FREE Full text][Medline][CrossRef]

51. Pellegrini CA, Steglitz J, Johnston W, Warnick J, Adams T, McFadden HG, Siddique J, Hedeker D, Spring B. Design and protocol of a randomized multiple behavior change trial: Make Better Choices 2 (MBC2). Contemp Clin Trials 2015 Mar; 41:85-92[FREE Full text][Medline][CrossRef]
52. Agboola S, Palacholla RS, Centi A, Kvedar J, Jethwani K. A Multimodal mHealth Intervention (FeatForward) to Improve Physical Activity Behavior in Patients with High Cardiometabolic Risk Factors: Rationale and Protocol for a Randomized Controlled Trial. JMIR Res Protoc 2016 May 12; 5(2):e84[FREE Full text][Medline][CrossRef]
53. Amorim AB, Pappas E, Simic M, Ferreira ML, Tiedemann A, Jennings M, Ferreira PH. Integrating Mobile health and Physical Activity to reduce the burden of Chronic low back pain Trial (IMPACT): a pilot trial protocol. BMC Musculoskelet Disord 2016; 17:36[FREE Full text][Medline][CrossRef]
54. Duncan MJ, Vandelanotte C, Trost SG, Rebar AL, Rogers N, Burton NW, Murawski B, Rayward A, Fenton S, Brown WJ. Balanced: a randomised trial examining the efficacy of two self-monitoring methods for an app-based multi-behaviour intervention to improve physical activity, sitting and sleep in adults. BMC Public Health 2016 Jul 30; 16:670[FREE Full text][Medline][CrossRef]
55. Jones D, Skrepnik N, Toselli RM, Leroy B. Incorporating novel mobile health technologies into management of knee osteoarthritis in patients treated with intra-articular hyaluronic acid: rationale and protocol of a randomized controlled trial. JMIR Res Protoc 2016 Aug 09;5(3):e164 [FREE Full text][Medline] [CrossRef]
56. Ortiz AM, Tueller SJ, Cook SL, Furberg RD. ActiviTeen: A Protocol for Deployment of a Consumer Wearable Device in an Academic Setting. JMIR Res Protoc 2016 Jul; 5(3):e153[FREE Full text][Medline][CrossRef]
57. Shin DW, Joh H, Yun JM, Kwon HT, Lee H, Min H, Shin J, Chung WJ, Park JH, Cho B. Design and baseline characteristics of participants in the Enhancing Physical Activity and Reducing Obesity through Smartcare and Financial Incentives (EPAROSFI): A pilot randomized controlled trial. Contemp Clin Trials 2016 Mar; 47:115-22[Medline][CrossRef]
58. Taylor D, Murphy J, Ahmad M, Purkayastha S, Scholtz S, Ramezani R, Vlaev I, Blakemore AIF, Darzi A. Quantified-Self for Obesity: Physical Activity Behaviour Sensing to Improve Health Outcomes. Stud Health Technol Inform 2016; 220:414-6[Medline]
59. van Nassau F, van der Ploeg HP, Abrahamsen F, Andersen E, Anderson AS, Bosmans JE, Bunn C, Chalmers M, Clissmann C, Gill JMR, Gray CM, Hunt K, Jelsma JG, La Guardia JG, Lemyre PN, Loudon DW, Macaulay L, Maxwell DJ, McConnachie A, Martin A, Mourselas N, Mutrie N, Nijhuis-van der Sanden R, O'Brien K, Pereira HV, Philpott M, Roberts GC, Rooksby J, Rost M, Røynesdal Ø, Sattar N, Silva MN, Sorensen M, Teixeira PJ, Treweek S, van Achterberg T, van de Glind I, van Mechelen W, Wyke S. Study protocol of European Fans in Training (EuroFIT): a four-country randomised controlled trial of a lifestyle program for men delivered in elite football clubs. BMC Public Health 2016 Jul 19; 16(1)[CrossRef]
60. Brickwood K, Smith ST, Watson G, Williams AD. The effect of ongoing feedback on physical activity levels following an exercise intervention in older adults: a randomised controlled trial protocol. BMC Sports Sci Med Rehabil 2017 Jan 10; 9(1)[CrossRef]
61. Ridgers ND, Timperio A, Brown H, Ball K, Macfarlane S, Lai SK, Richards K, Ngan W, Salmon J. A cluster-randomised controlled trial to promote physical activity in adolescents: the Raising Awareness of Physical Activity (RAW-PA) Study. BMC Public Health 2017 Jan 4; 17(1)[CrossRef]
62. Wolk S, Meißner T, Linke S, Müssle B, Wierick A, Bogner A, Sturm D, Rahbari NN, Distler M, Weitz J, Welsch T. Use of activity tracking in major visceral surgery—the Enhanced Perioperative Mobilization (EPM) trial: study protocol for a randomized controlled trial. Trials 2017 Feb 21; 18(1)[CrossRef]

63. Slootmaker SM, Chin A Paw MJ, Schuit AJ, Seidell JC, van Mechelen W. Promoting physical activity using an activity monitor and a tailored web-based advice: design of a randomized controlled trial [ISRCTN93896459]. BMC Public Health 2005 Dec 15; 5(1)[CrossRef]
64. Slootmaker SM, Chinapaw MJM, Schuit AJ, Seidell JC, Van MW. Feasibility and effectiveness of online physical activity advice based on a personal activity monitor: randomized controlled trial. J Med Internet Res 2009; 11(3):e27[FREE Full text][Medline][CrossRef]
65. Fujiki Y, Kazakos K, Puri C, Pavlidis I, Starren J, Levine J, editors. NEAT-o-games: ubiquitous activity-based gaming. In: CHI'07 extended abstracts. 2007. Presented at: CHI '07 Conference on Human Factors in Computing Systems 2007; Apr 30 - May 3; San Jose, CA.[CrossRef]
66. Hurling R, Catt M, Boni MD, Fairley BW, Hurst T, Murray P, Richardson A, Sodhi JS. Using internet and mobile phone technology to deliver an automated physical activity program: randomized controlled trial. J Med Internet Res 2007 Apr; 9(2):e7[FREE Full text][Medline][CrossRef]
67. Polzien KM, Jakicic JM, Tate DF, Otto AD. The efficacy of a technology-based system in a short-term behavioral weight loss intervention. Obesity (Silver Spring) 2007 Apr; 15(4):825-30[Medline][CrossRef]
68. Consolvo S, Klasnja P, McDonald D, Avrahami D, Froehlich J, LeGrand L. Flowers or a robot army? Encouraging awareness & activity with personal, mobile displays. 2008. Presented at: 10th International Conference on Ubiquitous Computing (UBICOMP) 2008; Sep 21-24; Florence, Italy.[CrossRef]
69. Faridi Z, Liberti L, Shuval K, Northrup V, Ali A, Katz DL. Evaluating the impact of mobile telephone technology on type 2 diabetic patients' self-management: the NICHE pilot study. J Eval Clin Pract 2008 Jun; 14(3):465-9[Medline][CrossRef]
70. Fujiki Y, Kazakos K, Puri C, Buddharaju P, Pavlidis I, Levine J. NEAT-o-Games. Comput. Entertain 2008 Jul 01; 6(2):21[CrossRef]
71. Goris A, Holmes R. The effect of a lifestyle activity intervention program on improving physical activity behavior of employees. 2008. Presented at: Third International Conference on Persuasive Technology (PERSUASIVE) 2008; June 4-6; Oulu, Finland.[CrossRef]
72. Lacroix J, Saini P, Holmes R. The relationship between goal difficulty performance in the context of a physical activity intervention program. 2008. Presented at: 10th International Conference on Human Computer Interaction with mobile devices and services 2008; Sep 2-5; Amsterdam, NL.[CrossRef]
73. Bickmore TW, Mauer D, Brown T. Context Awareness in a Handheld Exercise Agent. Pervasive Mob Comput 2009 Jun 01; 5(3):226-235[FREE Full text][Medline][CrossRef]
74. Fialho A, van den Heuval H, Shahab Q, Liu Q, Li L, Saini P. ActiveShare: sharing challenges to increase physical activities. In: CHI'09 Extended Abstracts. 2009. Presented at: CHI'09 Conference on Human Factors in Computing Systems 2009; Apr 4-9; Boston, MA.[CrossRef]
75. Arsand E, Tatara N, Østengen G, Hartvigsen G. Mobile phone-based self-management tools for type 2 diabetes: the few touch application. J Diabetes Sci Technol 2010 Mar; 4(2):328-36[FREE Full text][Medline]
76. Mattila E, Pärkkä J, Hermersdorf M, Kaasinen J, Vainio J, Samposalo K, Merilahti J, Kolari J, Kulju M, Lappalainen R, Korhonen I. Mobile diary for wellness management--results on usage and usability in two user studies. IEEE Trans Inf Technol Biomed 2008 Jul; 12(4):501-12[Medline][CrossRef]
77. Mattila E, Lappalainen R, Pärkkä J, Salminen J, Korhonen I. Use of a mobile phone diary for observing weight management and related behaviours. J Telemed Telecare 2010; 16(5):260-4[Medline][CrossRef]
78. Leal Penados A, Gielen M, Stappers P, Jongert T. Get up and move: an interactive cuddly toy that stimulates physical activity. Pers Ubiquit Comput 2009 Dec 17; 14(5):397-406[CrossRef]

79. Lim B, Shick A, Harrison C, Hudson S. Pediluma: Motivating Physical Activity Through Contextual Information and Social Influence. 2011. Presented at: 4th International Conference on Tangible, Embedded and Embodied Interaction (TEI) 2011; Jan 22-26; Funchal, Portugal.[CrossRef]
80. Shuger SL, Barry VW, Sui X, McClain A, Hand GA, Wilcox S, Meriwether RA, Hardin JW, Blair SN. Electronic feedback in a diet- and physical activity-based lifestyle intervention for weight loss: a randomized controlled trial. Int J Behav Nutr Phys Act 2011 May 18; 8:41[FREE Full text][Medline][CrossRef]
81. Burns P, Lueg C, Berkovsky S. Activmon: encouraging physical activity through ambient social awareness. In: CHI'12 Extended Abstracts. 2012. Presented at: CHI'12 Conference on Human Factors in Computing Systems 2012; May 5-10; Austin, TX.[CrossRef]
82. Pellegrini CA, Verba SD, Otto AD, Helsel DL, Davis KK, Jakicic JM. The comparison of a technology-based system and an in-person behavioral weight loss intervention. Obesity (Silver Spring) 2012 Feb; 20(2):356-63[FREE Full text][Medline][CrossRef]
83. Reijonsaari K, Vehtari A, Kahilakoski O, van MW, Aro T, Taimela S. The effectiveness of physical activity monitoring and distance counseling in an occupational setting - results from a randomized controlled trial (CoAct). BMC Public Health 2012; 12:344[FREE Full text][Medline][CrossRef]
84. Van HK, Boen F, Lefevre J. The effects of physical activity feedback on behavior and awareness in employees: study protocol for a randomized controlled trial. Int J Telemed Appl 2012; 2012:460712[FREE Full text][Medline][CrossRef]
85. Xu Y, Poole E, Miller A, Eiriksdottir E, Catrambone R, Mynatt E. Designing pervasive health games for sustainability, adaptability and sociability. 2012. Presented at: International Conference on the Foundations of Digital Games 2012; May 29 - Jun 1; Raleigh, NC.[CrossRef]
86. Poole E, Eiríksdóttir E, Miller A, Xu Y, Catrambone R, Mynatt E. Designing for spectators and coaches: social support in pervasive health games for youth. 2013. Presented at: 7th International Conference on Pervasive Computing Technologies for Healthcare (PervasiveHealth) 2013; May 5-8; Venice, Italy. [CrossRef]
87. Barwais FA, Cuddihy TF, Tomson LM. Physical activity, sedentary behavior and total wellness changes among sedentary adults: a 4-week randomized controlled trial. Health Qual Life Outcomes 2013; 11:183[FREE Full text][Medline][CrossRef]
88. Bentley F, Tollmar K, Stephenson P, Levy L, Jones B, Robertson S, Price E, Catrambone R, Wilson J. Health Mashups. ACM Trans. Comput.-Hum. Interact 2013 Nov 01; 20(5):1-27[CrossRef]
89. Chatterjee S, Byun J, Pottathil A, Moore M, Dutta K, Xie H. Persuasive sensing: a novel in-home monitoring technology to assist elderly adult diabetic patients. 2012. Presented at: 7th International Conference on Persuasive Technology (PERSUASIVE). Design for Health and Safety 2012; Jun 6-8; Linköping, Sweden.[CrossRef]
90. Chatterjee S, Dutta K, Xie H, Jongbok B, Pottathil A, Moore M. Persuasive and pervasive sensing: A new frontier to monitor, track and assist older adults suffering from type-2 diabetes. 2013. Presented at: 46th Hawaii International Conference on System Sciences (HICSS) 2013; Jan 7-10; Maui, Hawaii. [CrossRef]
91. Fitzsimons CF, Kirk A, Baker G, Michie F, Kane C, Mutrie N. Using an individualised consultation and activPAL™ feedback to reduce sedentary time in older Scottish adults: results of a feasibility and pilot study. Prev Med 2013 Nov; 57(5):718-20[Medline][CrossRef]
92. Harries T, Eslambolchilar P, Stride C, Rettie R, Walton S. Walking in the wild - using an always-on smartphone application to increase physical activity. 2013. Presented at: 14th IFIP International Conference on Human-Computer Interaction INTERACT 2013; Sep 2-6; Cape Town, South Africa. [CrossRef]

93. Harries T, Eslambolchilar P, Rettie R, Stride C, Walton S, van WHC. Effectiveness of a smartphone app in increasing physical activity amongst male adults: a randomised controlled trial. BMC Public Health 2016 Sept 02; 16:925[FREE Full text][Medline][CrossRef]
94. Hirano S, Farrell R, Danis C, Kellogg W. WalkMinder: encouraging an active lifestyle using mobile phone interruptions. In: CHI'13 Extended Abstracts. 2013. Presented at: CHI'13 Conference on Human Factors in Computing Systems 2013; Apr 27 - May 2; Paris, France.[CrossRef]
95. Khalil A, Abdallah S. Harnessing social dynamics through persuasive technology to promote healthier lifestyle. Computers in Human Behavior 2013 Nov; 29(6):2674-2681[CrossRef]
96. Khan A, Lee S. Need for a Context-Aware Personalized Health Intervention System to Ensure Long-Term Behavior Change to Prevent Obesity. 2013. Presented at: 35th International Conference on Software Engineering (ICSE), 5th International Workshop on Software Engineering in Health Care (SEHC) 2013; May 18-26; San Francisco, CA. p. 71-4[CrossRef]
97. King AC, Hekler EB, Grieco LA, Winter SJ, Sheats JL, Buman MP, Banerjee B, Robinson TN, Cirimele J. Harnessing different motivational frames via mobile phones to promote daily physical activity and reduce sedentary behavior in aging adults. PLoS One 2013 Apr; 8(4):e62613[FREE Full text][Medline][CrossRef]
98. King AC, Hekler EB, Grieco LA, Winter SJ, Sheats JL, Buman MP, Banerjee B, Robinson TN, Cirimele J. Effects of Three Motivationally Targeted Mobile Device Applications on Initial Physical Activity and Sedentary Behavior Change in Midlife and Older Adults: A Randomized Trial. PLoS One 2016; 11(6):e0156370[FREE Full text][Medline][CrossRef]
99. Nakajima T, Lehdonvirta V. Designing motivation using persuasive ambient mirrors. Pers Ubiquit Comput 2011 Oct 4; 17(1):107-126[CrossRef]
100. Tabak M, op den Akker H, Hermens H. Motivational cues as real-time feedback for changing daily activity behavior of patients with COPD. Patient Education and Counseling 2014 Mar; 94(3):372-378[CrossRef]
101. Tabak M, Vollenbroek-Hutten MM, van DVPD, van DPJ, Hermens HJ. A telerehabilitation intervention for patients with Chronic Obstructive Pulmonary Disease: a randomized controlled pilot trial. Clin Rehabil 2014 Jun; 28(6):582-91[Medline][CrossRef]
102. Valentin G, Howard A. Dealing with childhood obesity: passive versus active activity monitoring approaches for engaging individuals in exercise. 2013. Presented at: ISSNIP Biosignals and Biorobotics Conference (BRC) 2013; Feb 18-20; Rio de Janeiro, Brazil. p. 166-70[CrossRef]
103. Bond DS, Thomas JG, Raynor HA, Moon J, Sieling J, Trautvetter J, Leblond T, Wing RR. B-MOBILE--a smartphone-based intervention to reduce sedentary time in overweight/obese individuals: a within-subjects experimental trial. PLoS One 2014; 9(6):e100821[FREE Full text][Medline][CrossRef]
104. Thomas JG, Bond DS. Behavioral response to a just-in-time adaptive intervention (JITAI) to reduce sedentary behavior in obese adults: Implications for JITAI optimization. Health Psychol 2015 Dec; 34S:1261-7[FREE Full text][Medline][CrossRef]
105. Caulfield B, Kaljo I, Donnelly S. Use of a consumer market activity monitoring and feedback device improves exercise capacity and activity levels in COPD. Conf Proc IEEE Eng Med Biol Soc 2014; 2014:1765-8[Medline][CrossRef]
106. Chen Y, Pu P. HealthyTogether: exploring social incentives for mobile fitness applications. 2014. Presented at: 2nd International Symposium of Chinese CHI 2014; Apr 26-27; Toronto, ON, Canada. [CrossRef]
107. Glynn LG, Hayes PS, Casey M, Glynn F, Alvarez-Iglesias A, Newell J, Ólaighin G, Heaney D, Murphy AW. SMART MOVE - a smartphone-based intervention to promote physical activity in primary care: study protocol for a randomized controlled trial. Trials 2013; 14:157[FREE Full text][Medline][CrossRef]

108. Glynn LG, Hayes PS, Casey M, Glynn F, Alvarez-Iglesias A, Newell J, OLaighin G, Heaney D, O'Donnell M, Murphy AW. Effectiveness of a smartphone application to promote physical activity in primary care: the SMART MOVE randomised controlled trial. Br J Gen Pract 2014 Jul; 64(624):e384-91[Medline] [CrossRef]
109. Casey M, Hayes PS, Glynn F, OLaighin G, Heaney D, Murphy AW, Glynn LG. Patients' experiences of using a smartphone application to increase physical activity: the SMART MOVE qualitative study in primary care. Br J Gen Pract 2014 Aug; 64(625):e500-8[FREE Full text][Medline][CrossRef]
110. Miller A, Mynatt E. StepStream: a school-based pervasive social fitness system for everyday adolescent health. 2014. Presented at: CHI '14 Conference on Human Factors in Computing Systems 2014; Apr 26 - May 1; Toronto, ON, Canada.[CrossRef]
111. Thompson WG, Kuhle CL, Koepp GA, McCrady-Spitzer SK, Levine JA. "Go4Life" exercise counseling, accelerometer feedback, and activity levels in older people. Arch Gerontol Geriatr 2014 May; 58(3):314-9[Medline][CrossRef]
112. Thorndike AN, Mills S, Sonnenberg L, Palakshappa D, Gao T, Pau CT, Regan S. Activity monitor intervention to promote physical activity of physicians-in-training: randomized controlled trial. PLoS One 2014 Jun; 9(6):e100251[FREE Full text][Medline][CrossRef]
113. Verwey R, van der Weegen S, Spreeuwenberg M, Tange H, van der Weijden T, de Witte L. A pilot study of a tool to stimulate physical activity in patients with COPD or type 2 diabetes in primary care. J Telemed Telecare 2014 Jan 10; 20(1):29-34[CrossRef]
114. Walsh G, Golbeck J. A preliminary investigation of a personal informatics-based social game on behavior change. In: CHI'14 Extended Abstracts. 2014. Presented at: CHI'14 Conference on Human Factors in Computing Systems 2014; Apr 26 - May 1; Toronto, ON, Canada.[CrossRef]
115. Zuckerman O, Gal-Oz A. Deconstructing gamification: evaluating the effectiveness of continuous measurement, virtual rewards, and social comparison for promoting physical activity. Pers Ubiquit Comput 2014 Jul 5; 18(7):1705-1719[CrossRef]
116. Cadmus-Bertram LA, Marcus BH, Patterson RE, Parker BA, Morey BL. Randomized Trial of a Fitbit-Based Physical Activity Intervention for Women. Am J Prev Med 2015 Sept; 49(3):414-8[Medline][CrossRef]
117. Cadmus-Bertram L, Marcus BH, Patterson RE, Parker BA, Morey BL. Use of the Fitbit to Measure Adherence to a Physical Activity Intervention Among Overweight or Obese, Postmenopausal Women: Self-Monitoring Trajectory During 16 Weeks. JMIR Mhealth Uhealth 2015; 3(4):e96[FREE Full text] [Medline][CrossRef]
118. Direito A, Jiang Y, Whittaker R, Maddison R. Apps for IMproving FITness and Increasing Physical Activity Among Young People: The AIMFIT Pragmatic Randomized Controlled Trial. J Med Internet Res 2015 Aug 27; 17(8):e210[FREE Full text][Medline][CrossRef]
119. Finkelstein EA, Sahasranaman A, John G, Haaland BA, Bilger M, Sloan RA, Nang EEK, Evenson KR. Design and baseline characteristics of participants in the TRial of Economic Incentives to Promote Physical Activity (TRIPPA): a randomized controlled trial of a six month pedometer program with financial incentives. Contemp Clin Trials 2015 Mar; 41:238-47[Medline][CrossRef]
120. Finkelstein EA, Haaland BA, Bilger M, Sahasranaman A, Sloan RA, Nang EEK, Evenson KR. Effectiveness of activity trackers with and without incentives to increase physical activity (TRIPPA): a randomised controlled trial. The Lancet Diabetes & Endocrinology 2016 Dec; 4(12):983-995[CrossRef]
121. Frederix I, Van DN, Hansen D, Berger J, Bonne K, Alders T, Dendale P. Increasing the medium-term clinical benefits of hospital-based cardiac rehabilitation by physical activity telemonitoring in coronary artery disease patients. Eur J Prev Cardiol 2015 Feb; 22(2):150-8[Medline][CrossRef]
122. Frederix I, Hansen D, Coninx K, Vandervoort P, Vandijck D, Hens N, Van CE, Van DN, Dendale P. Medium-Term Effectiveness of a Comprehensive Internet-Based and Patient-Specific Telerehabilitation Program

With Text Messaging Support for Cardiac Patients: Randomized Controlled Trial. J Med Internet Res 2015; 17(7):e185[FREE Full text][Medline][CrossRef]
123. Garde A, Umedaly A, Abulnaga SM, Robertson L, Junker A, Chanoine JP, Ansermino JM, Dumont GA. Assessment of a Mobile Game ("MobileKids Monster Manor") to Promote Physical Activity Among Children. Games Health J 2015 Apr; 4(2):149-58[Medline][CrossRef]
124. Gouveia R, Karapanos E, Hassenzahl M. How do we engage with activity trackers?: A longitudinal study of Habito. 2015. Presented at: ACM International Joint Conference on Pervasive and Ubiquitous Computing (UBICOMP) 2015; Sep 7-11; Osaka, Japan.[CrossRef]
125. Guthrie N, Bradlyn A, Thompson SK, Yen S, Haritatos J, Dillon F, Cole SW. Development of an accelerometer-linked online intervention system to promote physical activity in adolescents. PLoS One 2015 May; 10(5):e0128639[FREE Full text][Medline][CrossRef]
126. Komninos A, Dunlop M, Rowe D, Hewitt A, Coull S. Using Degraded Music Quality to Encourage a Health Improving Walking Pace: BeatClearWalker. 2015. Presented at: 9th International Conference on Pervasive Computing Technologies for Healthcare (PervasiveHealth) 2015; May 20-23; Istanbul, Turkey. p. 57-64[CrossRef]
127. Lee M, Kim J, Forlizzi J, Kiesler S. Personalization revisited: a reflective approach helps people better personalize health services and motivates them to increase physical activity. 2015. Presented at: ACM International Joint Conference on Pervasive and Ubiquitous Computing (UBICOMP) 2015; Sep 7-11; Osaka, Japan.[CrossRef]
128. Lee M, Cha S, Nam T. Patina engraver: Visualizing activity logs as patina in fashionable trackers. 2015. Presented at: CHI '15 Conference on Human Factors in Computing Systems 2015; Apr 18-23; Seoul, Korea.[CrossRef]
129. Martin SS, Feldman DI, Blumenthal RS, Jones SR, Post WS, McKibben RA, Michos ED, Ndumele CE, Ratchford EV, Coresh J, Blaha MJ. mActive: A Randomized Clinical Trial of an Automated mHealth Intervention for Physical Activity Promotion. J Am Heart Assoc 2015 Nov 09; 4(11)[FREE Full text][Medline][CrossRef]
130. Munson S, Krupka E, Richardson C, Resnick P. Effects of public commitments and accountability in a technology-supported physical activity intervention. 2015. Presented at: CHI'15 Conference on Human Factors in Computing Systems 2015; Apr 18-23; Seoul, Korea.[CrossRef]
131. Rabbi M, Aung M, Zhang M, Choudhury T. MyBehavior: Automatic Personalized Health Feedback from User Behaviors and Preferences using Smartphones. 2015. Presented at: Proceedings of the ACM International Joint Conference on Pervasive and Ubiquitous Computing (UBICOMP) 2015; Sep 7-11; Osaka, Japan.[CrossRef]
132. Verwey R, van DWS, Spreeuwenberg M, Tange H, van DWT, de WL. A monitoring and feedback tool embedded in a counselling protocol to increase physical activity of patients with COPD or type 2 diabetes in primary care: study protocol of a three-arm cluster randomised controlled trial. BMC Fam Pract 2014; 15:93[FREE Full text][Medline][CrossRef]
133. van der Weegen S, Verwey R, Spreeuwenberg M, Tange H, van der Weijde T, de Witte L. It's LiFe! Mobile and Web-Based Monitoring and Feedback Tool Embedded in Primary Care Increases Physical Activity: A Cluster Randomized Controlled Trial. J Med Internet Res 2015 Jul 24; 17(7):e184[FREE Full text][Medline][CrossRef]
134. Verwey R, van der Weegan S, Spreeuwenberg M, Tange H, Van der Weijden T, De Witte L. Process evaluation of physical activity counselling with and without the use of mobile technology: A mixed methods study. Int J Nurs Stud 2016 Jan; 53:3-16[Medline][CrossRef]

135. Wadhwa R, Chugh A, Kumar A, Singh M, Yadav K, Eswaran S. SenseX: DesignDeployment of a Pervasive Wellness Monitoring Platform for Workplaces. 2015. Presented at: 13th International Conference on Service-Oriented Computing (ICSOC) 2015; Nov 16-19; Goa, India. p. 427-43[CrossRef]
136. Wang JB, Cadmus-Bertram LA, Natarajan L, White MM, Madanat H, Nichols JF, Ayala GX, Pierce JP. Wearable Sensor/Device (Fitbit One) and SMS Text-Messaging Prompts to Increase Physical Activity in Overweight and Obese Adults: A Randomized Controlled Trial. Telemed J E Health 2015 Oct; 21(10):782-92[Medline][CrossRef]
137. Watson S, Woodside JV, Ware LJ, Hunter SJ, McGrath A, Cardwell CR, Appleton KM, Young IS, McKinley MC. Effect of a Web-Based Behavior Change Program on Weight Loss and Cardiovascular Risk Factors in Overweight and Obese Adults at High Risk of Developing Cardiovascular Disease: Randomized Controlled Trial. J Med Internet Res 2015; 17(7):e177[FREE Full text][Medline][CrossRef]
138. Broekhuizen K, de GJ, Wijsman CA, Wijsman LW, Westendorp RGJ, Verhagen E, Slagboom PE, de CAJ, van MW, van HD, van DOF, Mooijaart SP. An Internet-Based Physical Activity Intervention to Improve Quality of Life of Inactive Older Adults: A Randomized Controlled Trial. J Med Internet Res 2016; 18(4):e74[FREE Full text][Medline][CrossRef]
139. Butryn ML, Arigo D, Raggio GA, Colasanti M, Forman EM. Enhancing physical activity promotion in midlife women with technology-based self-monitoring and social connectivity: A pilot study. J Health Psychol 2014 Dec 8;[Medline][CrossRef]
140. Choi J, Lee JH, Vittinghoff E, Fukuoka Y. mHealth Physical Activity Intervention: A Randomized Pilot Study in Physically Inactive Pregnant Women. Matern Child Health J 2016 May; 20(5):1091-101[Medline][CrossRef]
141. Ciman M, Donini M, Gaggi O, Aiolli F. Stairstep recognition and counting in a serious Game for increasing users' physical activity. Pers Ubiquit Comput 2016 Sept 22; 20(6):1015-1033[CrossRef]
142. Darvall JN, Parker A, Story DA. Feasibility and acceptability of remotely monitored pedometer-guided physical activity. Anaesth Intensive Care 2016 Jul; 44(4):501-6[FREE Full text][Medline]
143. Ding X, Xu J, Wang H, Chen G, Thind H, Zhang Y. WalkMore: Promoting Walking with Just-in-Time Context-Aware Prompts. 2016. Presented at: IEEE Wireless Health (WH) 2016; Oct 25-27; Bethesda, MD. p. 65-72[CrossRef]
144. Fennell C, Gerhart H, Seo Y, Hauge K, Glickman EL. Combined incentives versus no-incentive exercise programs on objectively measured physical activity and health-related variables. Physiol Behav 2016 Sept 01; 163:245-250[Medline][CrossRef]
145. Garde A, Umedaly A, Abulnaga SM, Junker A, Chanoine JP, Johnson M, Ansermino JM, Dumont GA. Evaluation of a Novel Mobile Exergame in a School-Based Environment. Cyberpsychol Behav Soc Netw 2016 Mar; 19(3):186-92[Medline][CrossRef]
146. Gilson ND, Pavey TG, Vandelanotte C, Duncan MJ, Gomersall SR, Trost SG, Brown WJ. Chronic disease risks and use of a smartphone application during a physical activity and dietary intervention in Australian truck drivers. Aust N Z J Public Health 2016 Feb; 40(1):91-3[Medline][CrossRef]
147. Glance D, Ooi E, Berman Y, Glance C, Barrett P. Impact of a Digital Activity Tracker-based Workplace Activity Program on Health and Wellbeing. 2016. Presented at: DH'16: Proceedings of 6th International Digital Health Conference 2016; Apr 11-13; Montreal, Québec, Canada. p. 37-41[CrossRef]
148. H-Jennings F, Clément M, Brown M, Leong B, Shen L, Dong C. Promote Students' Healthy Behavior Through Sensor and Game: A Randomized Controlled Trial. Med.Sci.Educ 2016 May 3; 26(3):349-355[CrossRef]
149. Hartman SJ, Nelson SH, Cadmus-Bertram LA, Patterson RE, Parker BA, Pierce JP. Technology- and Phone-Based Weight Loss Intervention: Pilot RCT in Women at Elevated Breast Cancer Risk. Am J Prev Med 2016 Nov; 51(5):714-721[Medline][CrossRef]

150. Herrmanny K, Ziegler J, Dogangün A. Supporting Users in Setting Effective Goals in Activity Tracking. 2016. Presented at: 11th International Conference on Persuasive Technology (PERSUASIVE) 2016; Apr 5-7; Salzburg, Austria. p. 11-26[CrossRef]
151. Melton BF, Buman MP, Vogel RL, Harris BS, Bigham LE. Wearable Devices to Improve Physical Activity and Sleep. Journal of Black Studies 2016 Jul 27; 47(6):610-625[CrossRef]
152. Patel MS, Asch DA, Rosin R, Small DS, Bellamy SL, Eberbach K, Walters KJ, Haff N, Lee SM, Wesby L, Hoffer K, Shuttleworth D, Taylor DH, Hilbert V, Zhu J, Yang L, Wang X, Volpp KG. Individual Versus Team-Based Financial Incentives to Increase Physical Activity: A Randomized, Controlled Trial. J Gen Intern Med 2016 Jul; 31(7):746-54[FREE Full text][Medline][CrossRef]
153. Patel MS, Volpp KG, Rosin R, Bellamy SL, Small DS, Fletcher MA, Osman-Koss R, Brady JL, Haff N, Lee SM, Wesby L, Hoffer K, Shuttleworth D, Taylor DH, Hilbert V, Zhu J, Yang L, Wang X, Asch DA. A Randomized Trial of Social Comparison Feedback and Financial Incentives to Increase Physical Activity. Am J Health Promot 2016 Jul; 30(6):416-24[Medline][CrossRef]
154. Patel MS, Asch DA, Rosin R, Small DS, Bellamy SL, Heuer J, Sproat S, Hyson C, Haff N, Lee SM, Wesby L, Hoffer K, Shuttleworth D, Taylor DH, Hilbert V, Zhu J, Yang L, Wang X, Volpp KG. Framing Financial Incentives to Increase Physical Activity Among Overweight and Obese Adults: A Randomized, Controlled Trial. Ann Intern Med 2016 Mar 15; 164(6):385-94[Medline][CrossRef]
155. Paul L, Wyke S, Brewster S, Sattar N, Gill JMR, Alexander G, Rafferty D, McFadyen AK, Ramsay A, Dybus A. Increasing physical activity in stroke survivors using STARFISH, an interactive mobile phone application: a pilot study. Top Stroke Rehabil 2016 Jun; 23(3):170-7[Medline][CrossRef]
156. Quintiliani LM, Mann DM, Puputti M, Quinn E, Bowen DJ. Pilot and Feasibility Test of a Mobile Health-Supported Behavioral Counseling Intervention for Weight Management Among Breast Cancer Survivors. JMIR Cancer 2016; 2(1)[FREE Full text][Medline][CrossRef]
157. Vorrink SNW, Kort HSM, Troosters T, Zanen P, Lammers JJ. Efficacy of an mHealth intervention to stimulate physical activity in COPD patients after pulmonary rehabilitation. Eur Respir J 2016 Oct; 48(4):1019-1029[Medline][CrossRef]
158. Walsh JC, Corbett T, Hogan M, Duggan J, McNamara A. An mHealth Intervention Using a Smartphone App to Increase Walking Behavior in Young Adults: A Pilot Study. JMIR Mhealth Uhealth 2016 Sept 22; 4(3):e109[FREE Full text][Medline][CrossRef]
159. Yingling LR, Brooks AT, Wallen GR, Peters-Lawrence M, McClurkin M, Cooper-McCann R, Wiley KL, Mitchell V, Saygbe JN, Johnson TD, Curry RKE, Johnson AA, Graham AP, Graham LA, Powell-Wiley TM. Community Engagement to Optimize the Use of Web-Based and Wearable Technology in a Cardiovascular Health and Needs Assessment Study: A Mixed Methods Approach. JMIR Mhealth Uhealth 2016 Apr 25; 4(2):e38[FREE Full text][Medline][CrossRef]
160. Ashton LM, Morgan PJ, Hutchesson MJ, Rollo ME, Collins CE. Feasibility and preliminary efficacy of the 'HEYMAN' healthy lifestyle program for young men: a pilot randomised controlled trial. Nutr J 2017 Jan 13; 16(1):2[FREE Full text][Medline][CrossRef]
161. Chen Y, Chen Y, Randriambelonoro M, Geissbuhler A, Pu P. Design Considerations for Social Fitness Applications: Comparing Chronically Ill Patients and Healthy Adults. 2017. Presented at: Proceedings of the 2017 ACM Conference on Computer Supported Cooperative Work and Social Computing (CSCW) 2017; Feb 25 - Mar 1; Portland, OR. p. 1753-62[CrossRef]
162. Chung AE, Skinner AC, Hasty SE, Perrin EM. Tweeting to Health: A Novel mHealth Intervention Using Fitbits and Twitter to Foster Healthy Lifestyles. Clin Pediatr (Phila) 2016 Jun 16;[Medline][CrossRef]
163. Gell NM, Grover KW, Humble M, Sexton M, Dittus K. Efficacy, feasibility, and acceptability of a novel technology-based intervention to support physical activity in cancer survivors. Support Care Cancer 2017 Apr; 25(4):1291-1300[Medline][CrossRef]

164. McMahon SK, Lewis B, Oakes JM, Wyman JF, Guan W, Rothman AJ. Assessing the Effects of Interpersonal and Intrapersonal Behavior Change Strategies on Physical Activity in Older Adults: a Factorial Experiment. Ann Behav Med 2017 Jun; 51(3):376-390[Medline][CrossRef]
165. Neil-Sztramko SE, Gotay CC, Sabiston CM, Demers PA, Campbell KC. Feasibility of a telephone and web-based physical activity intervention for women shift workers. Transl Behav Med 2017 Jun; 7(2):268-276[FREE Full text][Medline][CrossRef]
166. Valle CG, Deal AM, Tate DF. Preventing weight gain in African American breast cancer survivors using smart scales and activity trackers: a randomized controlled pilot study. J Cancer Surviv 2017 Feb; 11(1):133-148[Medline][CrossRef]
