# Supplementary material for: Evaluating the Impact of Physical Activity Apps and Wearables: Interdisciplinary Review
Source: JMIR Mhealth Uhealth. 2018 Mar 23;6(3):e58. doi: 10.2196/mhealth.9054 (PMC5889496; doi:10.2196/mhealth.9054)
Supplement: Multimedia Appendix 3 [file mhealth_v6i3e58_app3.pdf]

Multimedia Appendix 3: Research designs used in included studies and objectives investigated

Study	Research design	Objectives Investigated			
		Effectiveness[a]	Engagement	Acceptability	Usability
Protocols					
Walters et al 2010 [41]	2-group RCT	✓			
Kharrazi et al 2011 [42]	Nonrandomized group design	✓		✓	
Pellegrini et al 2012 [43]	3-group RCT	✓	✓		
Jimenez Garcia et al 2013 [44]	Repeated measures; randomized crossover design	✓		✓	
Geraedts et al 2014 [45]	Repeated measures; pre-post design	✓	✓		
Recio-Rodriguez et al 2014 [46]	2-group RCT	✓	✓		
Clayton et al 2015 [47]	2-group RCT	✓	✓		
Cooper et al 2015 [48]	4-group RCT	✓	✓	✓	
Granado-Font et al 2015 [49]	2-group RCT	✓	✓	✓	✓
Hurley et al 2015 [50]	4-group RCT	✓			
Pellegrini et al 2015 [51]	3-group RCT	✓			
Agboola et al 2016 [52]	2-group RCT	✓	✓	✓	
Amorim et al 2016 [53]	2-group RCT	✓		✓	
Duncan et al 2016 [54]	2-group RCT	✓	✓	✓	✓
Jones et al 2016 [55]	2-group RCT	✓	✓	✓	
Ortiz et al 2016 [56]	Observational		✓		

Shin et al 2016 [57]	3-group RCT	✓	✓	✓	
Taylor et al 2016[58]	Observational		✓	✓	
van Nassau et al 2016 [59]	2-group RCT	✓	✓	✓	
Brickwood et al 2017 [60]	2-group RCT	✓	✓		
Ridgers et al 2017 [61]	2-group RCT	✓	✓	✓	
Wolk et al 2017 [62]	2-group RCT	✓	✓		
Completed Trials					
Slootmaker et al 2005 [63,64]	2-group RCT	✓	✓		
Fujiki et al 2007 [65]	Case study with baseline phase	✓ (D)		✓	✓
Hurling et al 2007 [66]	2-group RCT	✓	✓		
Polzien et al 2007 [67]	2-group RCT	✓	✓		
Consolvo et al 2008 [68]	3-group RCT	✓		✓	
Faridi et al 2008 [69]	2-group RCT	✓	✓	✓	✓
Fujiki et al 2008 [70]	Nonrandomized group design	✓ (D)		✓	✓
Lacroix et al 2008 [71,72]	Repeated measures; pre-post design	✓			
Albaina et al 2009 [39]	Case study with baseline phase	✓ (D)		✓	✓
Bickmore et al 2009 [73]	Repeated measures; randomized crossover design	✓		✓	
Fialho et al 2009 [74]	Nonrandomized group design	✓		✓	

Arsand et al 2010 [75]	Case study	✓	✓		
Mattila et al 2010 [76,77]	Repeated measures; pre-post design	✓	✓	✓	
Penados et al 2010 [78]	Case study	✓ (D)		✓	
Lim et al 2011 [79]	4-group RCT	✓		✓	✓
Shuger et al 2011 [80]	4-group RCT	✓			
Burns et al 2012 [81]	Case study with baseline phase	✓ (D)	✓	✓	✓
Gomes et al 2012 [40]	Repeated measures; pre-post design	✓			
Pellegrini et al 2012 [82]	2-group RCT	✓	✓		
Reijonsaari et al 2012 [83]	2-group RCT	✓	✓		
Van Hoye et al 2012 [84]	4-group RCT	✓			
Xu, Poole, et al 2012 [85,86]	Repeated measures; longitudinal design	✓	✓	✓	
Barwais et al 2013 [87]	2-group RCT	✓			
Bentley et al 2013 [88]	Nonrandomized group design	✓	✓	✓	
Chatterjee et al 2013 [89,90]	Case study with baseline phase	✓ (D)		✓	
Fitzsimmons et al 2013 [91]	Repeated measures; pre-post design	✓			
Harries et al 2013 [92, 93]	2-group RCT	✓	✓	✓	
Hirano et al 2013 [94]	2-group RCT	✓		✓	
Khalil & Abdallah 2013 [95]	Nonrandomized group design	✓		✓	
Khan & Lee 2013 [96]	Case study with	✓ (D)		✓	

	baseline phase				
King et al 2013 [97,98]	4-group RCT	✓	✓	✓	
Nakajima et al 2013 [99]	Nonrandomized group design	✓		✓	
Tabak et al 2013 [101]	2-group RCT	✓	✓		
Valentin & Howard 2013 [102]	Repeated measures; cross-over design	✓ (D)			
Bond et al 2014 [103] [104]	Repeated measures; randomized crossover design	✓	✓	✓	
Caulfield et al 2014 [105]	Repeated measures; pre-post design	✓			
Chen & Pu 2014 [106]	3-group RCT	✓	✓	✓	
Glynn et al 2014 [107,108] [109]	2-group RCT	✓		✓	
Miller et al 2014 [110]	Repeated measures; longitudinal design	✓	✓	✓	
Thompson et al 2014 [111]	2-group RCT	✓			
Thorndike et al 2014 [112]	2-group RCT	✓			
Verwey et al 2014 [113]	Repeated measures; pre-post design	✓	✓	✓	✓
Walsh et al 2014 [114]	Repeated measures; randomized crossover design	✓			
Zuckerman et al 2014 [115]	3-group RCT	✓	✓		
Cadmus-Bertram et al 2015 [116, 117]	2-group RCT	✓	✓	✓	✓
Direito et al 2015 [118]	3-group RCT	✓	✓	✓	

Finkelstein et al 2015 [119,120]	4-group RCT	✓	✓		
Frederix et al 2015 [121]	2-group RCT	✓			
Frederix et al 2015 [122]	2-group RCT	✓		✓	✓
Garde et al 2015 [123]	Repeated measures; randomized crossover design	✓	✓	✓	
Gouveia et al 2015 [124]	Observational	✓	✓		
Guthrie et al 2015 [125]	3-group RCT	✓	✓		
Komninos et al 2015 [126]	Repeated measures; randomized crossover design	✓		✓	
Lee et al 2015 [127]	4-group RCT	✓		✓	
Lee et al 2015 [128]	Case study; with baseline phase	✓ (D)		✓	
Martin et al 2015 [129]	2-group RCT	✓		✓	
Munson et al 2015 [130]	3-group RCT	✓		✓	
Rabbi et al 2015 [131]	Single -case Design	✓			
Verwey et al 2015 [132-134]	2-group RCT	✓	✓	✓	
Wadwha et al 2015 [135]	Observational	✓ (D)	✓	✓	
Wang et al 2015 [136]	2-group RCT	✓	✓	✓	
Watson et al 2015 [137]	2-group RCT	✓	✓	✓	
Broekhuizen 2016 [138]	2-group RCT	✓			
Butryn et al 2016 [139]	Repeated measures; longitudinal design	✓	✓	✓	
Choi et al 2016 [140]	2-group RCT	✓	✓		

Ciman et al 2016 [141]	Repeated measures; randomized crossover design	✓ (D)	✓	✓	
Darvall et al 2016 [142]	Case study		✓	✓	✓
Ding et al 2016 [143]	2-group RCT	✓ (D)		✓	✓
Fennell et al 2016 [144]	Repeated measures; crossover design	✓			
Garde et al 2016 [145]	Nonrandomized group design	✓	✓	✓	✓
Gilson et al 2016 [146]	Repeated measures; longitudinal design		✓		
Glance et al 2016 [147]	Nonrandomized group design	✓			
H-Jennings et al 2016 [148]	3-group RCT	✓			
Hartman et al 2016 [149]	2-group RCT	✓			
Herrmany et al 2016 [150]	3-group RCT	✓		✓	
Melton et al 2016 [151]	2-group RCT	✓			
Patel et al 2016 [152-154]	4-group RCT	✓			
Paul et al 2016 [155]	Nonrandomized group design	✓			
Quintiliani et al 2016 [156]	Repeated measures; pre-post design	✓ (D)	✓	✓	
Vorrink et al 2016 [157]	2-group RCT	✓	✓		
Walsh et al 2016 [158]	Repeated measures; randomized crossover design	✓			
Yingling et al 2016 [159]	Case study		✓	✓	✓

Ashton et al 2017 [160]	2-group RCT	✓	✓	✓	✓
Chen et al 2017 [161]	Nonrandomized group design	✓		✓	
Chung et al 2017 [162]	Nonrandomized group design	✓ (D)	✓	✓	
Gell et al 2017 [163]	Repeated measures; pre-post design	✓		✓	
McMahon et al 2017 [164]	MOST	✓			
Neil-Sztramko et al 2017 [165]	Repeated measures; pre-post design	✓	✓	✓	
Valle et al 2017 [166]	3-group RCT	✓	✓	✓	
Total		**107**	**58**	**64**	**16**

[a]D: The study described (as opposed to statistically analyzed) effectiveness/impact, using descriptive statistics or visual analysis.

References

39. Albaina I, Visser T, van DMC, Vastenburg M. Flowie: A persuasive virtual coach to motivate elderly individuals to walk. 2009. Presented at: 3rd International Conference on Pervasive Computing Technologies for Healthcare (PervasiveHealth) 2009; Apr 1-3; London.[CrossRef]
40. Gomes N, Merugu D, O'Brien G, Mandayam C, Jia SY, Atikoglu B. Steptacular: An incentive mechanism for promoting wellness. 2012. Presented at: 4th International Conference on Communication Systems and Networks (COMSNETS) 2012; Jan 3-7; Bangalore.[CrossRef]
41. Walters DL, Sarela A, Fairfull A, Neighbour K, Cowen C, Stephens B, Sellwood T, Sellwood B, Steer M, Aust M, Francis R, Lee C, Hoffman S, Brealey G, Karunanithi M. A mobile phone-based care model for outpatient cardiac rehabilitation: the care assessment platform (CAP). BMC Cardiovasc Disord 2010; 10:5[FREE Full text][Medline][CrossRef]
42. Kharrazi H, Vincz L. Increasing physical activity by implementing a behavioral change intervention using pervasive personal health record system: an exploratory study. 2011. Presented at: 6th International Conference on Universal Access in Human-Computer Interaction (UAHCI). HCI International 2011; Jul 9-14; Orlando, FL.[CrossRef]
43. Pellegrini CA, Duncan JM, Moller AC, Buscemi J, Sularz A, DeMott A, Pictor A, Pagoto S, Siddique J, Spring B. A smartphone-supported weight loss program: design of the ENGAGED randomized controlled trial. BMC Public Health 2012; 12:1041[FREE Full text][Medline][CrossRef]
44. Jimenez GJ, Romero N, Keyson D, Havinga P, editors. ESTHER 1.3: integrating in-situ prompts to trigger self-reflection of physical activity in knowledge workers. 2013. Presented at:

ChileCHI'13 Chilean Conference on Human-Computer Interaction 2013; Nov 11-15; Temuco, Chile.[CrossRef]
45. Geraedts HAE, Zijlstra W, Zhang W, Bulstra S, Stevens M. Adherence to and effectiveness of an individually tailored home-based exercise program for frail older adults, driven by mobility monitoring: design of a prospective cohort study. BMC Public Health 2014; 14:570[FREE Full text][Medline][CrossRef]
46. Recio-Rodríguez JI, Martín-Cantera C, González-Viejo N, Gómez-Arranz A, Arietaleanizbeascoa MS, Schmolling-Guinovart Y, Maderuelo-Fernandez JA, Pérez-Arechaederra D, Rodriguez-Sanchez E, Gómez-Marcos MA, García-Ortiz L. Effectiveness of a smartphone application for improving healthy lifestyles, a randomized clinical trial (EVIDENT II): study protocol. BMC Public Health 2014; 14:254[FREE Full text][Medline][CrossRef]
47. Clayton C, Feehan L, Goldsmith CH, Miller WC, Grewal N, Ye J, Yoo JY, Li LC. Feasibility and preliminary efficacy of a physical activity counseling intervention using Fitbit in people with knee osteoarthritis: the TRACK-OA study protocol. Pilot Feasibility Stud 2015 Aug; 1:30[FREE Full text][Medline][CrossRef]
48. Cooper Andrew J M, Dearnley Katie, Williams Kate M, Sharp Stephen J, van Sluijs Esther M F, Brage Soren, Sutton Stephen, Griffin Simon J. Protocol for Get Moving: a randomised controlled trial to assess the effectiveness of three minimal contact interventions to promote fitness and physical activity in working adults. BMC Public Health 2015 Mar 27; 15:296[FREE Full text][Medline][CrossRef]
49. Granado-Font E, Flores-Mateo G, Sorlí-Aguilar M, Montaña-Carreras X, Ferre-Grau C, Barrera-Uriarte M, Oriol-Colominas E, Rey-Reñones C, Caules I, Satué-Gracia E. Effectiveness of a Smartphone application and wearable device for weight loss in overweight or obese primary care patients: protocol for a randomised controlled trial. BMC Public Health 2015 Jun 04; 15:531[FREE Full text][Medline][CrossRef]
50. Hurley JC, Hollingshead KE, Todd M, Jarrett CL, Tucker WJ, Angadi SS, Adams MA. The Walking Interventions Through Texting (WalkIT) Trial: Rationale, Design, and Protocol for a Factorial Randomized Controlled Trial of Adaptive Interventions for Overweight and Obese, Inactive Adults. JMIR Res Protoc 2015 Sept 11; 4(3):e108[FREE Full text][Medline][CrossRef]
51. Pellegrini CA, Steglitz J, Johnston W, Warnick J, Adams T, McFadden HG, Siddique J, Hedeker D, Spring B. Design and protocol of a randomized multiple behavior change trial: Make Better Choices 2 (MBC2). Contemp Clin Trials 2015 Mar; 41:85-92[FREE Full text][Medline][CrossRef]
52. Agboola S, Palacholla RS, Centi A, Kvedar J, Jethwani K. A Multimodal mHealth Intervention (FeatForward) to Improve Physical Activity Behavior in Patients with High Cardiometabolic Risk Factors: Rationale and Protocol for a Randomized Controlled Trial. JMIR Res Protoc 2016 May 12; 5(2):e84[FREE Full text][Medline][CrossRef]
53. Amorim AB, Pappas E, Simic M, Ferreira ML, Tiedemann A, Jennings M, Ferreira PH. Integrating Mobile health and Physical Activity to reduce the burden of Chronic low back pain Trial (IMPACT): a pilot trial protocol. BMC Musculoskelet Disord 2016; 17:36[FREE Full text][Medline][CrossRef]
54. Duncan MJ, Vandelanotte C, Trost SG, Rebar AL, Rogers N, Burton NW, Murawski B, Rayward A, Fenton S, Brown WJ. Balanced: a randomised trial examining the efficacy of two self-monitoring methods for an app-based multi-behaviour intervention to improve physical activity, sitting and sleep in adults. BMC Public Health 2016 Jul 30; 16:670[FREE Full text][Medline][CrossRef]

55. Jones D, Skrepnik N, Toselli RM, Leroy B. Incorporating novel mobile health technologies into management of knee osteoarthritis in patients treated with intra-articular hyaluronic acid: rationale and protocol of a randomized controlled trial. JMIR Res Protoc 2016 Aug 09;5(3):e164 [FREE Full text][Medline] [CrossRef]
56. Ortiz AM, Tueller SJ, Cook SL, Furberg RD. ActiviTeen: A Protocol for Deployment of a Consumer Wearable Device in an Academic Setting. JMIR Res Protoc 2016 Jul; 5(3):e153[FREE Full text][Medline][CrossRef]
57. Shin DW, Joh H, Yun JM, Kwon HT, Lee H, Min H, Shin J, Chung WJ, Park JH, Cho B. Design and baseline characteristics of participants in the Enhancing Physical Activity and Reducing Obesity through Smartcare and Financial Incentives (EPAROSFI): A pilot randomized controlled trial. Contemp Clin Trials 2016 Mar; 47:115-22[Medline][CrossRef]
58. Taylor D, Murphy J, Ahmad M, Purkayastha S, Scholtz S, Ramezani R, Vlaev I, Blakemore AIF, Darzi A. Quantified-Self for Obesity: Physical Activity Behaviour Sensing to Improve Health Outcomes. Stud Health Technol Inform 2016; 220:414-6[Medline]
59. van Nassau F, van der Ploeg HP, Abrahamsen F, Andersen E, Anderson AS, Bosmans JE, Bunn C, Chalmers M, Clissmann C, Gill JMR, Gray CM, Hunt K, Jelsma JG, La Guardia JG, Lemyre PN, Loudon DW, Macaulay L, Maxwell DJ, McConnachie A, Martin A, Mourselas N, Mutrie N, Nijhuis-van der Sanden R, O'Brien K, Pereira HV, Philpott M, Roberts GC, Rooksby J, Rost M, Røynesdal Ø, Sattar N, Silva MN, Sorensen M, Teixeira PJ, Treweek S, van Achterberg T, van de Glind I, van Mechelen W, Wyke S. Study protocol of European Fans in Training (EuroFIT): a four-country randomised controlled trial of a lifestyle program for men delivered in elite football clubs. BMC Public Health 2016 Jul 19; 16(1)[CrossRef]
60. Brickwood K, Smith ST, Watson G, Williams AD. The effect of ongoing feedback on physical activity levels following an exercise intervention in older adults: a randomised controlled trial protocol. BMC Sports Sci Med Rehabil 2017 Jan 10; 9(1)[CrossRef]
61. Ridgers ND, Timperio A, Brown H, Ball K, Macfarlane S, Lai SK, Richards K, Ngan W, Salmon J. A cluster-randomised controlled trial to promote physical activity in adolescents: the Raising Awareness of Physical Activity (RAW-PA) Study. BMC Public Health 2017 Jan 4; 17(1)[CrossRef]
62. Wolk S, Meißner T, Linke S, Müssle B, Wierick A, Bogner A, Sturm D, Rahbari NN, Distler M, Weitz J, Welsch T. Use of activity tracking in major visceral surgery—the Enhanced Perioperative Mobilization (EPM) trial: study protocol for a randomized controlled trial. Trials 2017 Feb 21; 18(1)[CrossRef]
63. Slootmaker SM, Chin A Paw MJ, Schuit AJ, Seidell JC, van Mechelen W. Promoting physical activity using an activity monitor and a tailored web-based advice: design of a randomized controlled trial [ISRCTN93896459]. BMC Public Health 2005 Dec 15; 5(1)[CrossRef]
64. Slootmaker SM, Chinapaw MJM, Schuit AJ, Seidell JC, Van MW. Feasibility and effectiveness of online physical activity advice based on a personal activity monitor: randomized controlled trial. J Med Internet Res 2009; 11(3):e27[FREE Full text][Medline][CrossRef]
65. Fujiki Y, Kazakos K, Puri C, Pavlidis I, Starren J, Levine J, editors. NEAT-o-games: ubiquitous activity-based gaming. In: CHI'07 extended abstracts. 2007. Presented at: CHI '07 Conference on Human Factors in Computing Systems 2007; Apr 30 - May 3; San Jose, CA.[CrossRef]
66. Hurling R, Catt M, Boni MD, Fairley BW, Hurst T, Murray P, Richardson A, Sodhi JS. Using internet and mobile phone technology to deliver an automated physical activity program: randomized controlled trial. J Med Internet Res 2007 Apr; 9(2):e7[FREE Full text][Medline] [CrossRef]

67. Polzien KM, Jakicic JM, Tate DF, Otto AD. The efficacy of a technology-based system in a short-term behavioral weight loss intervention. Obesity (Silver Spring) 2007 Apr; 15(4):825-30[Medline][CrossRef]
68. Consolvo S, Klasnja P, McDonald D, Avrahami D, Froehlich J, LeGrand L. Flowers or a robot army? Encouraging awareness & activity with personal, mobile displays. 2008. Presented at: 10th International Conference on Ubiquitous Computing (UBICOMP) 2008; Sep 21-24; Florence, Italy.[CrossRef]
69. Faridi Z, Liberti L, Shuval K, Northrup V, Ali A, Katz DL. Evaluating the impact of mobile telephone technology on type 2 diabetic patients' self-management: the NICHE pilot study. J Eval Clin Pract 2008 Jun; 14(3):465-9[Medline][CrossRef]
70. Fujiki Y, Kazakos K, Puri C, Buddharaju P, Pavlidis I, Levine J. NEAT-o-Games. Comput. Entertain 2008 Jul 01; 6(2):21[CrossRef]
71. Goris A, Holmes R. The effect of a lifestyle activity intervention program on improving physical activity behavior of employees. 2008. Presented at: Third International Conference on Persuasive Technology (PERSUASIVE) 2008; June 4-6; Oulu, Finland.[CrossRef]
72. Lacroix J, Saini P, Holmes R. The relationship between goal difficulty performance in the context of a physical activity intervention program. 2008. Presented at: 10th International Conference on Human Computer Interaction with mobile devices and services 2008; Sep 2-5; Amsterdam, NL.[CrossRef]
73. Bickmore TW, Mauer D, Brown T. Context Awareness in a Handheld Exercise Agent. Pervasive Mob Comput 2009 Jun 01; 5(3):226-235[FREE Full text][Medline][CrossRef]
74. Fialho A, van den Heuval H, Shahab Q, Liu Q, Li L, Saini P. ActiveShare: sharing challenges to increase physical activities. In: CHI'09 Extended Abstracts. 2009. Presented at: CHI'09 Conference on Human Factors in Computing Systems 2009; Apr 4-9; Boston, MA.[CrossRef]
75. Arsand E, Tatara N, Østengen G, Hartvigsen G. Mobile phone-based self-management tools for type 2 diabetes: the few touch application. J Diabetes Sci Technol 2010 Mar; 4(2):328-36[FREE Full text][Medline]
76. Mattila E, Pärkkä J, Hermersdorf M, Kaasinen J, Vainio J, Samposalo K, Merilahti J, Kolari J, Kulju M, Lappalainen R, Korhonen I. Mobile diary for wellness management--results on usage and usability in two user studies. IEEE Trans Inf Technol Biomed 2008 Jul; 12(4):501-12[Medline][CrossRef]
77. Mattila E, Lappalainen R, Pärkkä J, Salminen J, Korhonen I. Use of a mobile phone diary for observing weight management and related behaviours. J Telemed Telecare 2010; 16(5):260-4[Medline][CrossRef]
78. Leal Penados A, Gielen M, Stappers P, Jongert T. Get up and move: an interactive cuddly toy that stimulates physical activity. Pers Ubiquit Comput 2009 Dec 17; 14(5):397-406[CrossRef]
79. Lim B, Shick A, Harrison C, Hudson S. Pediluma: Motivating Physical Activity Through Contextual Information and Social Influence. 2011. Presented at: 4th International Conference on Tangible, Embedded and Embodied Interaction (TEI) 2011; Jan 22-26; Funchal, Portugal.[CrossRef]
80. Shuger SL, Barry VW, Sui X, McClain A, Hand GA, Wilcox S, Meriwether RA, Hardin JW, Blair SN. Electronic feedback in a diet- and physical activity-based lifestyle intervention for weight loss: a randomized controlled trial. Int J Behav Nutr Phys Act 2011 May 18; 8:41[FREE Full text][Medline][CrossRef]
81. Burns P, Lueg C, Berkovsky S. Activmon: encouraging physical activity through ambient social awareness. In: CHI'12 Extended Abstracts. 2012. Presented at: CHI'12 Conference on Human Factors in Computing Systems 2012; May 5-10; Austin, TX.[CrossRef]

82. Pellegrini CA, Verba SD, Otto AD, Helsel DL, Davis KK, Jakicic JM. The comparison of a technology-based system and an in-person behavioral weight loss intervention. Obesity (Silver Spring) 2012 Feb; 20(2):356-63[FREE Full text][Medline][CrossRef]
83. Reijonsaari K, Vehtari A, Kahilakoski O, van MW, Aro T, Taimela S. The effectiveness of physical activity monitoring and distance counseling in an occupational setting - results from a randomized controlled trial (CoAct). BMC Public Health 2012; 12:344[FREE Full text] [Medline][CrossRef]
84. Van HK, Boen F, Lefevre J. The effects of physical activity feedback on behavior and awareness in employees: study protocol for a randomized controlled trial. Int J Telemed Appl 2012; 2012:460712[FREE Full text][Medline][CrossRef]
85. Xu Y, Poole E, Miller A, Eiriksdottir E, Catrambone R, Mynatt E. Designing pervasive health games for sustainability, adaptability and sociability. 2012. Presented at: International Conference on the Foundations of Digital Games 2012; May 29 - Jun 1; Raleigh, NC.[CrossRef]
86. Poole E, Eiríksdóttir E, Miller A, Xu Y, Catrambone R, Mynatt E. Designing for spectators and coaches: social support in pervasive health games for youth. 2013. Presented at: 7th International Conference on Pervasive Computing Technologies for Healthcare (PervasiveHealth) 2013; May 5-8; Venice, Italy.[CrossRef]
87. Barwais FA, Cuddihy TF, Tomson LM. Physical activity, sedentary behavior and total wellness changes among sedentary adults: a 4-week randomized controlled trial. Health Qual Life Outcomes 2013; 11:183[FREE Full text][Medline][CrossRef]
88. Bentley F, Tollmar K, Stephenson P, Levy L, Jones B, Robertson S, Price E, Catrambone R, Wilson J. Health Mashups. ACM Trans. Comput.-Hum. Interact 2013 Nov 01; 20(5):1-27[CrossRef]
89. Chatterjee S, Byun J, Pottathil A, Moore M, Dutta K, Xie H. Persuasive sensing: a novel in-home monitoring technology to assist elderly adult diabetic patients. 2012. Presented at: 7th International Conference on Persuasive Technology (PERSUASIVE). Design for Health and Safety 2012; Jun 6-8; Linköping, Sweden.[CrossRef]
90. Chatterjee S, Dutta K, Xie H, Jongbok B, Pottathil A, Moore M. Persuasive and pervasive sensing: A new frontier to monitor, track and assist older adults suffering from type-2 diabetes. 2013. Presented at: 46th Hawaii International Conference on System Sciences (HICSS) 2013; Jan 7-10; Maui, Hawaii.[CrossRef]
91. Fitzsimons CF, Kirk A, Baker G, Michie F, Kane C, Mutrie N. Using an individualised consultation and activPAL™ feedback to reduce sedentary time in older Scottish adults: results of a feasibility and pilot study. Prev Med 2013 Nov; 57(5):718-20[Medline][CrossRef]
92. Harries T, Eslambolchilar P, Stride C, Rettie R, Walton S. Walking in the wild - using an always-on smartphone application to increase physical activity. 2013. Presented at: 14th IFIP International Conference on Human-Computer Interaction INTERACT 2013; Sep 2-6; Cape Town, South Africa.[CrossRef]
93. Harries T, Eslambolchilar P, Rettie R, Stride C, Walton S, van WHC. Effectiveness of a smartphone app in increasing physical activity amongst male adults: a randomised controlled trial. BMC Public Health 2016 Sept 02; 16:925[FREE Full text][Medline][CrossRef]
94. Hirano S, Farrell R, Danis C, Kellogg W. WalkMinder: encouraging an active lifestyle using mobile phone interruptions. In: CHI'13 Extended Abstracts. 2013. Presented at: CHI'13 Conference on Human Factors in Computing Systems 2013; Apr 27 - May 2; Paris, France. [CrossRef]
95. Khalil A, Abdallah S. Harnessing social dynamics through persuasive technology to promote healthier lifestyle. Computers in Human Behavior 2013 Nov; 29(6):2674-2681[CrossRef]

96. Khan A, Lee S. Need for a Context-Aware Personalized Health Intervention System to Ensure Long-Term Behavior Change to Prevent Obesity. 2013. Presented at: 35th International Conference on Software Engineering (ICSE), 5th International Workshop on Software Engineering in Health Care (SEHC) 2013; May 18-26; San Francisco, CA. p. 71-4[CrossRef]
97. King AC, Hekler EB, Grieco LA, Winter SJ, Sheats JL, Buman MP, Banerjee B, Robinson TN, Cirimele J. Harnessing different motivational frames via mobile phones to promote daily physical activity and reduce sedentary behavior in aging adults. PLoS One 2013 Apr; 8(4):e62613[FREE Full text][Medline][CrossRef]
98. King AC, Hekler EB, Grieco LA, Winter SJ, Sheats JL, Buman MP, Banerjee B, Robinson TN, Cirimele J. Effects of Three Motivationally Targeted Mobile Device Applications on Initial Physical Activity and Sedentary Behavior Change in Midlife and Older Adults: A Randomized Trial. PLoS One 2016; 11(6):e0156370[FREE Full text][Medline][CrossRef]
99. Nakajima T, Lehdonvirta V. Designing motivation using persuasive ambient mirrors. Pers Ubiquit Comput 2011 Oct 4; 17(1):107-126[CrossRef]
100. Tabak M, op den Akker H, Hermens H. Motivational cues as real-time feedback for changing daily activity behavior of patients with COPD. Patient Education and Counseling 2014 Mar; 94(3):372-378[CrossRef]
101. Tabak M, Vollenbroek-Hutten MM, van DVPD, van DPJ, Hermens HJ. A telerehabilitation intervention for patients with Chronic Obstructive Pulmonary Disease: a randomized controlled pilot trial. Clin Rehabil 2014 Jun; 28(6):582-91[Medline][CrossRef]
102. Valentin G, Howard A. Dealing with childhood obesity: passive versus active activity monitoring approaches for engaging individuals in exercise. 2013. Presented at: ISSNIP Biosignals and Biorobotics Conference (BRC) 2013; Feb 18-20; Rio de Janeiro, Brazil. p. 166-70[CrossRef]
103. Bond DS, Thomas JG, Raynor HA, Moon J, Sieling J, Trautvetter J, Leblond T, Wing RR. B-MOBILE--a smartphone-based intervention to reduce sedentary time in overweight/obese individuals: a within-subjects experimental trial. PLoS One 2014; 9(6):e100821[FREE Full text][Medline][CrossRef]
104. Thomas JG, Bond DS. Behavioral response to a just-in-time adaptive intervention (JITAI) to reduce sedentary behavior in obese adults: Implications for JITAI optimization. Health Psychol 2015 Dec; 34S:1261-7[FREE Full text][Medline][CrossRef]
105. Caulfield B, Kaljo I, Donnelly S. Use of a consumer market activity monitoring and feedback device improves exercise capacity and activity levels in COPD. Conf Proc IEEE Eng Med Biol Soc 2014; 2014:1765-8[Medline][CrossRef]
106. Chen Y, Pu P. HealthyTogether: exploring social incentives for mobile fitness applications. 2014. Presented at: 2nd International Symposium of Chinese CHI 2014; Apr 26-27; Toronto, ON, Canada.[CrossRef]
107. Glynn LG, Hayes PS, Casey M, Glynn F, Alvarez-Iglesias A, Newell J, Ólaighin G, Heaney D, Murphy AW. SMART MOVE - a smartphone-based intervention to promote physical activity in primary care: study protocol for a randomized controlled trial. Trials 2013; 14:157[FREE Full text][Medline][CrossRef]
108. Glynn LG, Hayes PS, Casey M, Glynn F, Alvarez-Iglesias A, Newell J, OLaighin G, Heaney D, O'Donnell M, Murphy AW. Effectiveness of a smartphone application to promote physical activity in primary care: the SMART MOVE randomised controlled trial. Br J Gen Pract 2014 Jul; 64(624):e384-91[Medline][CrossRef]
109. Casey M, Hayes PS, Glynn F, OLaighin G, Heaney D, Murphy AW, Glynn LG. Patients' experiences of using a smartphone application to increase physical activity: the SMART MOVE

qualitative study in primary care. Br J Gen Pract 2014 Aug; 64(625):e500-8[FREE Full text][Medline][CrossRef]
110. Miller A, Mynatt E. StepStream: a school-based pervasive social fitness system for everyday adolescent health. 2014. Presented at: CHI '14 Conference on Human Factors in Computing Systems 2014; Apr 26 - May 1; Toronto, ON, Canada.[CrossRef]
111. Thompson WG, Kuhle CL, Koepp GA, McCrady-Spitzer SK, Levine JA. "Go4Life" exercise counseling, accelerometer feedback, and activity levels in older people. Arch Gerontol Geriatr 2014 May; 58(3):314-9[Medline][CrossRef]
112. Thorndike AN, Mills S, Sonnenberg L, Palakshappa D, Gao T, Pau CT, Regan S. Activity monitor intervention to promote physical activity of physicians-in-training: randomized controlled trial. PLoS One 2014 Jun; 9(6):e100251[FREE Full text][Medline][CrossRef]
113. Verwey R, van der Weegen S, Spreeuwenberg M, Tange H, van der Weijden T, de Witte L. A pilot study of a tool to stimulate physical activity in patients with COPD or type 2 diabetes in primary care. J Telemed Telecare 2014 Jan 10; 20(1):29-34[CrossRef]
114. Walsh G, Golbeck J. A preliminary investigation of a personal informatics-based social game on behavior change. In: CHI'14 Extended Abstracts. 2014. Presented at: CHI'14 Conference on Human Factors in Computing Systems 2014; Apr 26 - May 1; Toronto, ON, Canada.[CrossRef]
115. Zuckerman O, Gal-Oz A. Deconstructing gamification: evaluating the effectiveness of continuous measurement, virtual rewards, and social comparison for promoting physical activity. Pers Ubiquit Comput 2014 Jul 5; 18(7):1705-1719[CrossRef]
116. Cadmus-Bertram LA, Marcus BH, Patterson RE, Parker BA, Morey BL. Randomized Trial of a Fitbit-Based Physical Activity Intervention for Women. Am J Prev Med 2015 Sept; 49(3):414-8[Medline][CrossRef]
117. Cadmus-Bertram L, Marcus BH, Patterson RE, Parker BA, Morey BL. Use of the Fitbit to Measure Adherence to a Physical Activity Intervention Among Overweight or Obese, Postmenopausal Women: Self-Monitoring Trajectory During 16 Weeks. JMIR Mhealth Uhealth 2015; 3(4):e96[FREE Full text][Medline][CrossRef]
118. Direito A, Jiang Y, Whittaker R, Maddison R. Apps for IMproving FITness and Increasing Physical Activity Among Young People: The AIMFIT Pragmatic Randomized Controlled Trial. J Med Internet Res 2015 Aug 27; 17(8):e210[FREE Full text][Medline][CrossRef]
119. Finkelstein EA, Sahasranaman A, John G, Haaland BA, Bilger M, Sloan RA, Nang EEK, Evenson KR. Design and baseline characteristics of participants in the TRial of Economic Incentives to Promote Physical Activity (TRIPPA): a randomized controlled trial of a six month pedometer program with financial incentives. Contemp Clin Trials 2015 Mar; 41:238-47[Medline][CrossRef]
120. Finkelstein EA, Haaland BA, Bilger M, Sahasranaman A, Sloan RA, Nang EEK, Evenson KR. Effectiveness of activity trackers with and without incentives to increase physical activity (TRIPPA): a randomised controlled trial. The Lancet Diabetes & Endocrinology 2016 Dec; 4(12):983-995[CrossRef]
121. Frederix I, Van DN, Hansen D, Berger J, Bonne K, Alders T, Dendale P. Increasing the medium-term clinical benefits of hospital-based cardiac rehabilitation by physical activity telemonitoring in coronary artery disease patients. Eur J Prev Cardiol 2015 Feb; 22(2):150-8[Medline][CrossRef]
122. Frederix I, Hansen D, Coninx K, Vandervoort P, Vandijck D, Hens N, Van CE, Van DN, Dendale P. Medium-Term Effectiveness of a Comprehensive Internet-Based and Patient-Specific

Telerehabilitation Program With Text Messaging Support for Cardiac Patients: Randomized Controlled Trial. J Med Internet Res 2015; 17(7):e185[FREE Full text][Medline][CrossRef]
123. Garde A, Umedaly A, Abulnaga SM, Robertson L, Junker A, Chanoine JP, Ansermino JM, Dumont GA. Assessment of a Mobile Game ("MobileKids Monster Manor") to Promote Physical Activity Among Children. Games Health J 2015 Apr; 4(2):149-58[Medline][CrossRef]
124. Gouveia R, Karapanos E, Hassenzahl M. How do we engage with activity trackers?: A longitudinal study of Habito. 2015. Presented at: ACM International Joint Conference on Pervasive and Ubiquitous Computing (UBICOMP) 2015; Sep 7-11; Osaka, Japan.[CrossRef]
125. Guthrie N, Bradlyn A, Thompson SK, Yen S, Haritatos J, Dillon F, Cole SW. Development of an accelerometer-linked online intervention system to promote physical activity in adolescents. PLoS One 2015 May; 10(5):e0128639[FREE Full text][Medline][CrossRef]
126. Komninos A, Dunlop M, Rowe D, Hewitt A, Coull S. Using Degraded Music Quality to Encourage a Health Improving Walking Pace: BeatClearWalker. 2015. Presented at: 9th International Conference on Pervasive Computing Technologies for Healthcare (PervasiveHealth) 2015; May 20-23; Istanbul, Turkey. p. 57-64[CrossRef]
127. Lee M, Kim J, Forlizzi J, Kiesler S. Personalization revisited: a reflective approach helps people better personalize health services and motivates them to increase physical activity. 2015. Presented at: ACM International Joint Conference on Pervasive and Ubiquitous Computing (UBICOMP) 2015; Sep 7-11; Osaka, Japan.[CrossRef]
128. Lee M, Cha S, Nam T. Patina engraver: Visualizing activity logs as patina in fashionable trackers. 2015. Presented at: CHI '15 Conference on Human Factors in Computing Systems 2015; Apr 18-23; Seoul, Korea.[CrossRef]
129. Martin SS, Feldman DI, Blumenthal RS, Jones SR, Post WS, McKibben RA, Michos ED, Ndumele CE, Ratchford EV, Coresh J, Blaha MJ. mActive: A Randomized Clinical Trial of an Automated mHealth Intervention for Physical Activity Promotion. J Am Heart Assoc 2015 Nov 09; 4(11) [FREE Full text][Medline][CrossRef]
130. Munson S, Krupka E, Richardson C, Resnick P. Effects of public commitments and accountability in a technology-supported physical activity intervention. 2015. Presented at: CHI'15 Conference on Human Factors in Computing Systems 2015; Apr 18-23; Seoul, Korea. [CrossRef]
131. Rabbi M, Aung M, Zhang M, Choudhury T. MyBehavior: Automatic Personalized Health Feedback from User Behaviors and Preferences using Smartphones. 2015. Presented at: Proceedings of the ACM International Joint Conference on Pervasive and Ubiquitous Computing (UBICOMP) 2015; Sep 7-11; Osaka, Japan.[CrossRef]
132. Verwey R, van DWS, Spreeuwenberg M, Tange H, van DWT, de WL. A monitoring and feedback tool embedded in a counselling protocol to increase physical activity of patients with COPD or type 2 diabetes in primary care: study protocol of a three-arm cluster randomised controlled trial. BMC Fam Pract 2014; 15:93[FREE Full text][Medline][CrossRef]
133. van der Weegen S, Verwey R, Spreeuwenberg M, Tange H, van der Weijde T, de Witte L. It's LiFe! Mobile and Web-Based Monitoring and Feedback Tool Embedded in Primary Care Increases Physical Activity: A Cluster Randomized Controlled Trial. J Med Internet Res 2015 Jul 24; 17(7):e184[FREE Full text][Medline][CrossRef]
134. Verwey R, van der Weegan S, Spreeuwenberg M, Tange H, Van der Weijden T, De Witte L. Process evaluation of physical activity counselling with and without the use of mobile technology: A mixed methods study. Int J Nurs Stud 2016 Jan; 53:3-16[Medline][CrossRef]

135. Wadhwa R, Chugh A, Kumar A, Singh M, Yadav K, Eswaran S. SenseX: DesignDeployment of a Pervasive Wellness Monitoring Platform for Workplaces. 2015. Presented at: 13th International Conference on Service-Oriented Computing (ICSOC) 2015; Nov 16-19; Goa, India. p. 427-43[CrossRef]
136. Wang JB, Cadmus-Bertram LA, Natarajan L, White MM, Madanat H, Nichols JF, Ayala GX, Pierce JP. Wearable Sensor/Device (Fitbit One) and SMS Text-Messaging Prompts to Increase Physical Activity in Overweight and Obese Adults: A Randomized Controlled Trial. Telemed J E Health 2015 Oct; 21(10):782-92[Medline][CrossRef]
137. Watson S, Woodside JV, Ware LJ, Hunter SJ, McGrath A, Cardwell CR, Appleton KM, Young IS, McKinley MC. Effect of a Web-Based Behavior Change Program on Weight Loss and Cardiovascular Risk Factors in Overweight and Obese Adults at High Risk of Developing Cardiovascular Disease: Randomized Controlled Trial. J Med Internet Res 2015; 17(7):e177[FREE Full text][Medline][CrossRef]
138. Broekhuizen K, de GJ, Wijsman CA, Wijsman LW, Westendorp RGJ, Verhagen E, Slagboom PE, de CAJ, van MW, van HD, van DOF, Mooijaart SP. An Internet-Based Physical Activity Intervention to Improve Quality of Life of Inactive Older Adults: A Randomized Controlled Trial. J Med Internet Res 2016; 18(4):e74[FREE Full text][Medline][CrossRef]
139. Butryn ML, Arigo D, Raggio GA, Colasanti M, Forman EM. Enhancing physical activity promotion in midlife women with technology-based self-monitoring and social connectivity: A pilot study. J Health Psychol 2014 Dec 8;[Medline][CrossRef]
140. Choi J, Lee JH, Vittinghoff E, Fukuoka Y. mHealth Physical Activity Intervention: A Randomized Pilot Study in Physically Inactive Pregnant Women. Matern Child Health J 2016 May; 20(5):1091-101[Medline][CrossRef]
141. Ciman M, Donini M, Gaggi O, Aiolli F. Stairstep recognition and counting in a serious Game for increasing users' physical activity. Pers Ubiquit Comput 2016 Sept 22; 20(6):1015-1033[CrossRef]
142. Darvall JN, Parker A, Story DA. Feasibility and acceptability of remotely monitored pedometer-guided physical activity. Anaesth Intensive Care 2016 Jul; 44(4):501-6[FREE Full text][Medline]
143. Ding X, Xu J, Wang H, Chen G, Thind H, Zhang Y. WalkMore: Promoting Walking with Just-in-Time Context-Aware Prompts. 2016. Presented at: IEEE Wireless Health (WH) 2016; Oct 25-27; Bethesda, MD. p. 65-72[CrossRef]
144. Fennell C, Gerhart H, Seo Y, Hauge K, Glickman EL. Combined incentives versus no-incentive exercise programs on objectively measured physical activity and health-related variables. Physiol Behav 2016 Sept 01; 163:245-250[Medline][CrossRef]
145. Garde A, Umedaly A, Abulnaga SM, Junker A, Chanoine JP, Johnson M, Ansermino JM, Dumont GA. Evaluation of a Novel Mobile Exergame in a School-Based Environment. Cyberpsychol Behav Soc Netw 2016 Mar; 19(3):186-92[Medline][CrossRef]
146. Gilson ND, Pavey TG, Vandelanotte C, Duncan MJ, Gomersall SR, Trost SG, Brown WJ. Chronic disease risks and use of a smartphone application during a physical activity and dietary intervention in Australian truck drivers. Aust N Z J Public Health 2016 Feb; 40(1):91-3[Medline][CrossRef]
147. Glance D, Ooi E, Berman Y, Glance C, Barrett P. Impact of a Digital Activity Tracker-based Workplace Activity Program on Health and Wellbeing. 2016. Presented at: DH'16: Proceedings of 6th International Digital Health Conference 2016; Apr 11-13; Montreal, Québec, Canada. p. 37-41[CrossRef]

148. H-Jennings F, Clément M, Brown M, Leong B, Shen L, Dong C. Promote Students' Healthy Behavior Through Sensor and Game: A Randomized Controlled Trial. Med.Sci.Educ 2016 May 3; 26(3):349-355[CrossRef]
149. Hartman SJ, Nelson SH, Cadmus-Bertram LA, Patterson RE, Parker BA, Pierce JP. Technology- and Phone-Based Weight Loss Intervention: Pilot RCT in Women at Elevated Breast Cancer Risk. Am J Prev Med 2016 Nov; 51(5):714-721[Medline][CrossRef]
150. Herrmanny K, Ziegler J, Dogangün A. Supporting Users in Setting Effective Goals in Activity Tracking. 2016. Presented at: 11th International Conference on Persuasive Technology (PERSUASIVE) 2016; Apr 5-7; Salzburg, Austria. p. 11-26[CrossRef]
151. Melton BF, Buman MP, Vogel RL, Harris BS, Bigham LE. Wearable Devices to Improve Physical Activity and Sleep. Journal of Black Studies 2016 Jul 27; 47(6):610-625[CrossRef]
152. Patel MS, Asch DA, Rosin R, Small DS, Bellamy SL, Eberbach K, Walters KJ, Haff N, Lee SM, Wesby L, Hoffer K, Shuttleworth D, Taylor DH, Hilbert V, Zhu J, Yang L, Wang X, Volpp KG. Individual Versus Team-Based Financial Incentives to Increase Physical Activity: A Randomized, Controlled Trial. J Gen Intern Med 2016 Jul; 31(7):746-54[FREE Full text][Medline][CrossRef]
153. Patel MS, Volpp KG, Rosin R, Bellamy SL, Small DS, Fletcher MA, Osman-Koss R, Brady JL, Haff N, Lee SM, Wesby L, Hoffer K, Shuttleworth D, Taylor DH, Hilbert V, Zhu J, Yang L, Wang X, Asch DA. A Randomized Trial of Social Comparison Feedback and Financial Incentives to Increase Physical Activity. Am J Health Promot 2016 Jul; 30(6):416-24[Medline][CrossRef]
154. Patel MS, Asch DA, Rosin R, Small DS, Bellamy SL, Heuer J, Sproat S, Hyson C, Haff N, Lee SM, Wesby L, Hoffer K, Shuttleworth D, Taylor DH, Hilbert V, Zhu J, Yang L, Wang X, Volpp KG. Framing Financial Incentives to Increase Physical Activity Among Overweight and Obese Adults: A Randomized, Controlled Trial. Ann Intern Med 2016 Mar 15; 164(6):385-94[Medline][CrossRef]
155. Paul L, Wyke S, Brewster S, Sattar N, Gill JMR, Alexander G, Rafferty D, McFadyen AK, Ramsay A, Dybus A. Increasing physical activity in stroke survivors using STARFISH, an interactive mobile phone application: a pilot study. Top Stroke Rehabil 2016 Jun; 23(3):170-7[Medline][CrossRef]
156. Quintiliani LM, Mann DM, Puputti M, Quinn E, Bowen DJ. Pilot and Feasibility Test of a Mobile Health-Supported Behavioral Counseling Intervention for Weight Management Among Breast Cancer Survivors. JMIR Cancer 2016; 2(1)[FREE Full text][Medline][CrossRef]
157. Vorrink SNW, Kort HSM, Troosters T, Zanen P, Lammers JJ. Efficacy of an mHealth intervention to stimulate physical activity in COPD patients after pulmonary rehabilitation. Eur Respir J 2016 Oct; 48(4):1019-1029[Medline][CrossRef]
158. Walsh JC, Corbett T, Hogan M, Duggan J, McNamara A. An mHealth Intervention Using a Smartphone App to Increase Walking Behavior in Young Adults: A Pilot Study. JMIR Mhealth Uhealth 2016 Sept 22; 4(3):e109[FREE Full text][Medline][CrossRef]
159. Yingling LR, Brooks AT, Wallen GR, Peters-Lawrence M, McClurkin M, Cooper-McCann R, Wiley KL, Mitchell V, Saygbe JN, Johnson TD, Curry RKE, Johnson AA, Graham AP, Graham LA, Powell-Wiley TM. Community Engagement to Optimize the Use of Web-Based and Wearable Technology in a Cardiovascular Health and Needs Assessment Study: A Mixed Methods Approach. JMIR Mhealth Uhealth 2016 Apr 25; 4(2):e38[FREE Full text][Medline][CrossRef]
160. Ashton LM, Morgan PJ, Hutchesson MJ, Rollo ME, Collins CE. Feasibility and preliminary efficacy of the 'HEYMAN' healthy lifestyle program for young men: a pilot randomised controlled trial. Nutr J 2017 Jan 13; 16(1):2[FREE Full text][Medline][CrossRef]

161. Chen Y, Chen Y, Randriambelonoro M, Geissbuhler A, Pu P. Design Considerations for Social Fitness Applications: Comparing Chronically Ill Patients and Healthy Adults. 2017. Presented at: Proceedings of the 2017 ACM Conference on Computer Supported Cooperative Work and Social Computing (CSCW) 2017; Feb 25 - Mar 1; Portland, OR. p. 1753-62[CrossRef]
162. Chung AE, Skinner AC, Hasty SE, Perrin EM. Tweeting to Health: A Novel mHealth Intervention Using Fitbits and Twitter to Foster Healthy Lifestyles. Clin Pediatr (Phila) 2016 Jun 16; [Medline][CrossRef]
163. Gell NM, Grover KW, Humble M, Sexton M, Dittus K. Efficacy, feasibility, and acceptability of a novel technology-based intervention to support physical activity in cancer survivors. Support Care Cancer 2017 Apr; 25(4):1291-1300[Medline][CrossRef]
164. McMahon SK, Lewis B, Oakes JM, Wyman JF, Guan W, Rothman AJ. Assessing the Effects of Interpersonal and Intrapersonal Behavior Change Strategies on Physical Activity in Older Adults: a Factorial Experiment. Ann Behav Med 2017 Jun; 51(3):376-390[Medline][CrossRef]
165. Neil-Sztramko SE, Gotay CC, Sabiston CM, Demers PA, Campbell KC. Feasibility of a telephone and web-based physical activity intervention for women shift workers. Transl Behav Med 2017 Jun; 7(2):268-276[FREE Full text][Medline][CrossRef]
166. Valle CG, Deal AM, Tate DF. Preventing weight gain in African American breast cancer survivors using smart scales and activity trackers: a randomized controlled pilot study. J Cancer Surviv 2017 Feb; 11(1):133-148[Medline][CrossRef]
